# Integrated transcriptomic and immune enzymatic analyses uncover coordinated immunometabolic responses in large yellow croaker (*Larimichthys crocea*) to *Metanophrys* sp. infection

**DOI:** 10.3389/fimmu.2025.1636453

**Published:** 2025-07-16

**Authors:** Ruiling Zhou, Kangshuai Sun, Xiao Xie, Fei Yin, Jorge Galindo-Villegas

**Affiliations:** ^1^ School of Marine Sciences, National Demonstration Center for Experimental (Aquaculture) Education, Ningbo University, Ningbo, China; ^2^ Department of Genomics, Faculty of Biosciences and Aquaculture, Nord University, Bodo, Norway

**Keywords:** chemokine signaling pathway, host-parasite interactions, IL-17 signaling, immunometabolism, oxidative stress, skin transcriptome, FOXO1 (forkhead box O1), RAF1 gene

## Abstract

*Metanophrys* sp. (Scuticociliatida) has recently emerged as a significant parasitic threat in large yellow croaker (*Larimichthys crocea*) aquaculture. To elucidate the host response, we conducted an experimental infection followed by an integrated analysis combining immune enzymatic profiling and transcriptome sequencing. Antioxidant and immune enzyme activities, including SOD, CAT, and MDA in the skin, gill, and liver, and LYZ and Na^+^/K^+^-ATPase in the skin and gill were monitored from 0 to 72 hours post-infection (hpi). The results revealed tissue- and time-specific significant changes, indicating increased oxidative stress and activation of compensatory antioxidant and mucosal immune defenses. At the resolution phase of infection (72 hpi), dorsal skin tissue was subjected to RNA sequencing (RNA-seq), identifying 6,360 differentially expressed genes (DEGs), including 3,164 upregulated and 2,702 downregulated transcripts. GO and KEGG enrichment analyses revealed strong activation of key immune signaling pathways, such as *NOD-like receptor*, *IL-17*, *chemokine*, and *cytokine–cytokine receptor interaction*, alongside metabolic reprogramming involving *oxidative phosphorylation*, *glycolysis/gluconeogenesis*, and *lipid metabolism*. Inflammatory mediators associated with IL-17 signaling, including *cox2*, *cxcl8, mmp9* and *hsp90*, together with chemokine-related effectors such as *akt, raf1*, and *rhoA* were significantly upregulated, suggesting strong mucosal inflammation and thrombocyte involvement, functionally analogous to the platelet-activation pathway in mammals. Notably, immunometabolic convergence was evidenced by co-upregulation of genes such as *il1b*, *il6*, *cxcl8*, *mmp9*, *pnpla2*, and *foxo1*, reflecting the simultaneous activation of inflammatory and metabolic regulatory programs during host defense. Swiss-Prot annotations confirmed the conserved functional roles of these genes in cytokine signaling, energy mobilization, and tissue protection. qPCR validation of 12 representative genes showed strong concordance with the RNA-seq expression profile (*R²*=0.98). Together, these findings demonstrate that *L. crocea* mounts a temporally coordinated immunometabolic response to *Metanophrys* sp., providing mechanistic insights into mucosal defense and offering candidate biomarkers for targeted disease management in marine aquaculture.

## 1 Introduction

Aquaculture has expanded rapidly in recent decades to address the increasing global demand for sustainable sources of animal protein. Notably, in 2022, aquaculture production surpassed that of capture fisheries for the first time, contributing 51% of the total aquatic animal output (1). However, this growth has been accompanied by the intensification of farming practices, which in turn has increased the vulnerability of cultured species to emerging infectious agents, particularly opportunistic parasites (2).

Among these, scuticociliates (phylum Ciliophora, subclass Scuticociliatida) represent a group of facultative ectoparasites that can survive in organic-rich environments and shift to an invasive pathogenic lifestyle under conditions of host stress (3). These ciliates initially colonize epithelial surfaces such as the skin and gills, but can infiltrate deeper tissues, including blood vessels, visceral organs, and even the brain, where they induce extensive necrosis through direct cytophagy (4–6). Several scuticociliates, including *Porpostoma notata* (7) and *Miamiensis avidus* (8), have been identified as significant pathogens in marine fish, with earlier reports highlighting their broad host range and virulence potential (9, 10).

Recently, our groups identified a novel scuticociliate, *Metanophrys* sp., from diseased large yellow croaker (*Larimichthys crocea*), confirming its pathogenicity through experimental infection (11). While its taxonomy and ecological behavior have been approached (12), the pathophysiological effects of *Metanophrys* sp. on *L. crocea*, and the underlying immune defense mechanisms, remain largely uncharacterized.

*Larimichthys crocea* is a commercially important marine species in China (13), with an annual production of over 280,000 tons in 2023 (14). The rapid expansion of high-density farming has exacerbated disease outbreaks, with scuticociliate infections posing significant challenges (6, 11). Teleost fish rely on both innate and adaptive immune mechanisms to resist parasitic invasion. At mucosal barriers such as the skin and gills, immune-related enzymes play central roles in defending against parasites. Antioxidant enzymes, including superoxide dismutase (SOD) (15, 16) and catalase (CAT) (17, 18), mitigate oxidative stress by neutralizing reactive oxygen species (ROS), generated during phagocyte activity (19). Concurrently, lysozyme (LZM) contributes to antimicrobial activity (20), and Na^+^/K^+^-ATPase regulates osmoregulatory responses during gill parasitism (21). Oxidative stress markers, such as malondialdehyde (MDA), further reflect tissue damage, redox imbalance, and lipid peroxidation (22).

Transcriptomics technologies have advanced our understanding of host-parasite interactions in fish (23, 24). For example, RNA-seq of *Philasterides dicentrarchi*-infected turbot revealed early upregulation of pro-inflammatory cytokines such as *il1b* and *tnfa* in immune tissue (25). However, no previous studies have integrated enzymatic profiling with transcriptomic analysis to elucidate the coordinated response of *L. crocea* against scuticociliate infection.

Recent progress in vertebrate immunology has also highlighted the importance of immunometabolism defined as the interface between immune signaling and metabolic reprogramming (26). Cytokine signaling pathways such as IL-17, TNF, and MAPK not only regulate inflammation but also influence cellular bioenergetics, redox status, and immune cell activation. The relevance of such immunometabolic pathways in teleost immunity is increasingly recognized but remains poorly explored under parasitic infection. In this study, we investigated the immunometabolic response of *L. crocea* to *Metanophrys* sp. infection through an integrated approach combining enzymatic immune profiling and skin transcriptomic analysis. Specifically, we examined: (i) tissue-specific changes in immune-related enzymatic activities (SOD, CAT, LZM, Na+/K+-ATPase) and oxidative stress markers (MDA); (ii) differential gene expression and immune signaling pathway activation at 72 hours post-infection; and (iii) mechanistic links between immune enzyme activity and transcriptomic regulation of host defenses. Our findings provide novel insights into the dual role of innate immune effectors and immunometabolic pathways in shaping host resistance to parasitic protozoan infection in marine aquaculture and offer a scientific basis for the diagnosis and targeted treatment intervention of this disease.

## 2 Materials and methods

### 2.1 Ethical statement

All experimental procedures involving fish were reviewed and approved by the Institutional Animal Care and Use Committee (IACUC) of Ningbo University, China (0564/2023). The study adhered to all applicable national and institutional guidelines for the care and use of laboratory animals.

### 2.2 Fish and parasite sources

A total of 120 healthy large yellow croaker with an average body length of 9 ± 0.5 cm and 10 ± 3 g weight, were obtained from commercial marine cages in Yinzhou, Ningbo, China. Prior to the trial, fish were acclimated for 14 days in a recirculating aquaculture system (RAS) at the pilot base of Ningbo University. During acclimation and throughout the trial, water temperature was maintained at 20 ± 2°C and salinity at 30 ± 2‰, conditions considered optimal for the fish species and representative of typical *Metanophrys* sp. outbreak scenarios. Continuous aeration was provided. The pathogenic ciliate *Metanophrys* sp., previously isolated and monoclonally cultured in our laboratory (11), was used in all infection challenges.

### 2.3 Experimental design and infection protocol

**Figure 1:**
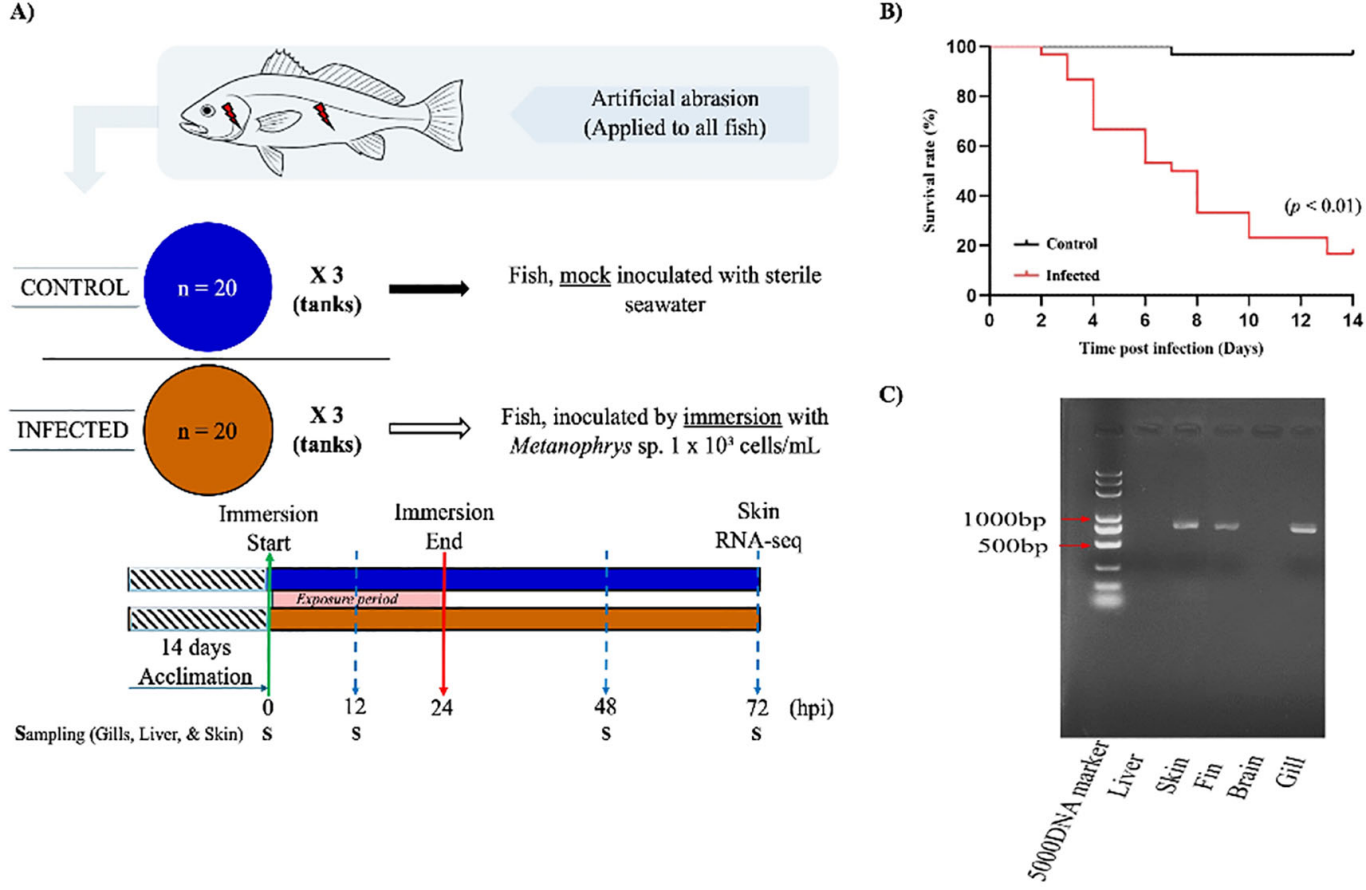
Experimental design and infection dynamics of *L. crocea* following exposure to *Metanophrys* sp. **(A)** Schematic overview of the experimental setup. After a 14-day acclimation, two groups of *L. crocea* (n=20/tank; three replicate tanks per group) were mechanically abraded on the gills and skin. The infected group was immersed in seawater containing *Metanophrys* sp. (1×10^3^ ciliates/mL), while the control group was mock inoculated with sterile seawater. Immersion lasted 24 hours, after which fish were returned to normal seawater. Tissue samples were collected at 0, 12, 24, 48, and 72 hpi for enzymatic assays, while additional skin from the same side of each fish were collected at 72 hpi for RNA-seq. **(B)** Kaplan–Meier survival curves of infected and control fish over 10 days. The infected group exhibited significantly higher cumulative mortality, reaching 80% by day 10 (*p*<0.01, log-rank test). **(C)** PCR detection of *Metanophrys* sp. in infected fish tissues. Parasite-specific amplicons were detected in the skin, fin, and gills, but not in the brain or liver. (S, sampling point).

Fish were randomly assigned to two treatment groups: control and infected (n= 60 fish/group). Each group was subdivided into three biological replicates (n=20 fish/100 L tank) under identical environmental conditions. To facilitate parasite attachment, fish in the infected group were gently abraded on the dorsal epidermis and operculum using a sterile scalpel. Control fish underwent the same abrasion procedure but were mock exposed (**Figure 1A**). The infected group was inoculated with *Metanophrys* sp. at a final concentration of 1 x 10³ cells/mL, while control fish received an equivalent volume of sterile seawater. After a 24-hours exposure period, all fish were transferred to *Metanophrys* sp. -free seawater, with 50% water renewal every 24 h. Fish were not feed during the experimental period. Clinical signs and mortality were monitored and recoded daily. Infection was confirmed by PCR amplification of gill and skin tissue using *Metanophrys*-specific mitochondrial COI- primers: F1 (5’-TTTTCGTTGTAGTTCCTG-3’) and R1 (5’-GACGATCTAAAGCCATCA-3’), following previously published protocols (11).

### 2.4 Sample collection and processing

At 0 h (pre-infection), 12, 24, 48, and 72 hpi, skin, gill, and liver tissues were collected from three randomly selected fish per tank (n=9/time point) for enzymatic assays. For transcriptomic analysis, skin samples from the same side of each fish were collected and flash-frozen in liquid nitrogen and stored at -80°C. These included samples from uninfected controls (C1–C3) and infected fish at 72 hpi (IN1–IN3).

### 2.5 Enzyme activity assays

Tissue samples were retrieved from storage at -80°C and homogenized for enzymatic analysis. For each 1g of *L. crocea* tissue (skin, gill, or liver), 9 mL of pre-chilled 0.9% physiological saline solution was added. Two sterile magnetic beads were included, and the samples were homogenized using a tissue grinder. The homogenates were then centrifuged at 3000 rpm for 10 minutes at 4°C. The resulting supernatants were transferred to clean Eppendorf tubes and stored at 4°C for subsequent assays. Enzyme activities were quantified using commercial kits from Nanjing Jiancheng Bioengineering Institute (Nanjing, China), following the manufacturer’s instructions. The assays included superoxide dismutase (SOD), catalase (CAT), malondialdehyde (MDA), lysozyme (LYZ), and Na^+^/K^+^- ATPase. Absorbance was measured using a microplate reader (AMR-100, Sinoocean, China).

### 2.6 RNA extraction and transcriptome sequencing

Total RNA was extracted from the skin of three pooled fish per group using TransZol reagent (TransGen Biotech, China). The quality, integrity, and purity of the RNA were assessed by 1.5% agarose gel electrophoresis, a NanoDrop spectrophotometer (Thermo Fisher, USA), and RNA Integrity Number (RIN) analysis using an Agilent 2100 Bioanalyzer (Agilent Technologies, USA). All RNA samples were stored at -80°C until future use. Samples that passed quality control, with RIN values ≥ 7.0 were sent to Wuhan Feisha Biotechnology Co., Ltd. (Wuhan, China) for cDNA library construction and high-throughput sequencing using the MGI platform (MGI Tech Co., Ltd., Shenzhen, China).

### 2.7 RNA-seq data processing and differential expression analysis

Raw sequencing reads were quality-filtered using SOAPnuke software (27). The resulting paired-end clean reads were aligned against the *L. crocea* reference genome (RefSeq: PRJNA245366), using HISAT2 (28). Bowtie2 (29) was employed to align the quality-controlled second-generation reads to the reference transcriptome, and transcript abundance was quantified with RSEM (30) the number of reads aligned to each transcript for each sample was normalized to fragments per kilobase of transcript per million mapped reads (FPKM) values. Differential gene expression analysis was performed using the DESeq R software package (31). Genes with a |Log2 fold change| > 1 or |Log2 fold change| < -1 and a false discovery rate (FDR) < 0.05 were considered significantly differentially expressed. Gene ontology (GO) and Kyoto Encyclopedia of Genes and Genomes (KEGG) enrichment analyses were subsequently conducted on the differentially expressed genes (DEGs). GO terms and KEGG pathways with Benjamini-Hochberg adjusted p-values *<* 0.05 were considered significantly enriched. To complement functional pathway enrichment, protein-level annotation was performed by mapping transcripts to the Swiss-Prot database (release 2023_03) using BLASTx with a minimum E-value threshold of 1e-5. Swiss-Prot accession numbers were retrieved for each DEG and used to support cross-species functional assignment and identification of immune and metabolic proteins.

### 2.8 Quantitative real-time PCR validation

Twelve immune-related DEGs were randomly selected for validation by qPCR using the 2× SYBR Green Premix Pro Taq HS qPCR Kit (Accurate Biology). Gene-specific primers (**Supplementary Table 1**) were designed with Primer 5 software, and β-actin was used as the internal reference gene. Total RNA was reverse transcribed into cDNA using the ReverTra Ace qPCR RT Master Mix (Toyobo, Japan), following the manufacturer’s instructions. PCR amplification was carried out in a total volume of 20 μL, consisting of 10 μL of 2× SYBR Green Pro Taq HS Premix, 0.4 μL each of forward and reverse primers, 0.5 μL of cDNA, and 8.7 μL of RNase-free water. The thermal cycling conditions were as follows: initial denaturation at 95°C for 5 min; followed by 40 cycles of 95°C for 10 s, 56°C for 10 s, and 72°C for 10 s. A melt curve analysis was performed at the end of the amplification to verify product specificity. Relative gene expression levels were calculated using the 2-ΔΔCt method (32), with amplification efficiency for each primer pair considered.

### 2.9 Statistical analysis

All data were analyzed using SPSS 26.0 (IBM Corp., Armonk, NY, USA) and Microsoft Excel. Before to statistical comparisons, data were tested for normality and homogeneity of variances. One-way ANOVA was performed where appropriate to assess overall differences among groups. Differences between control and infected groups were assessed using an independent sample t-test, with significance set at *p<*0.05. Results are presented as mean ± standard error (mean ± S.E.). Graphs were generated using GraphPad Prism software version 8.0 (GraphPad Software, San Diego, CA, USA).

## 3 Results

### 3.1 Artificial infection experiment

The artificial infection experiment revealed a progressive worsening of symptoms in *L. crocea* over time, culminating in mortality in severe cases. Fish exposed to *Metanophrys* sp. through abrasion-immersion exhibited a cumulative survival rate of 20% by day 10 post-infection (**Figure 1B**). However, a significant reduction in survival up to approximately 40% occurred between days 4-6, which was already markedly higher than that observed in the control group (*p* < 0.01). To assess parasite distribution, PCR analysis was conducted on gill filaments, skin, fins, brain, and liver. *Metanophrys* sp. was detected in the gills, skin, and fins of infected fish, whereas no parasite DNA was found in the brain or liver tissues (**Figure 1C**), suggesting that *Metanophrys* sp. invades the host through a localized colonization of external epithelial surfaces.

### 3.2 Effect of *Metanophrys* sp. infection on SOD activity in *L. crocea*

**Figure 2:**
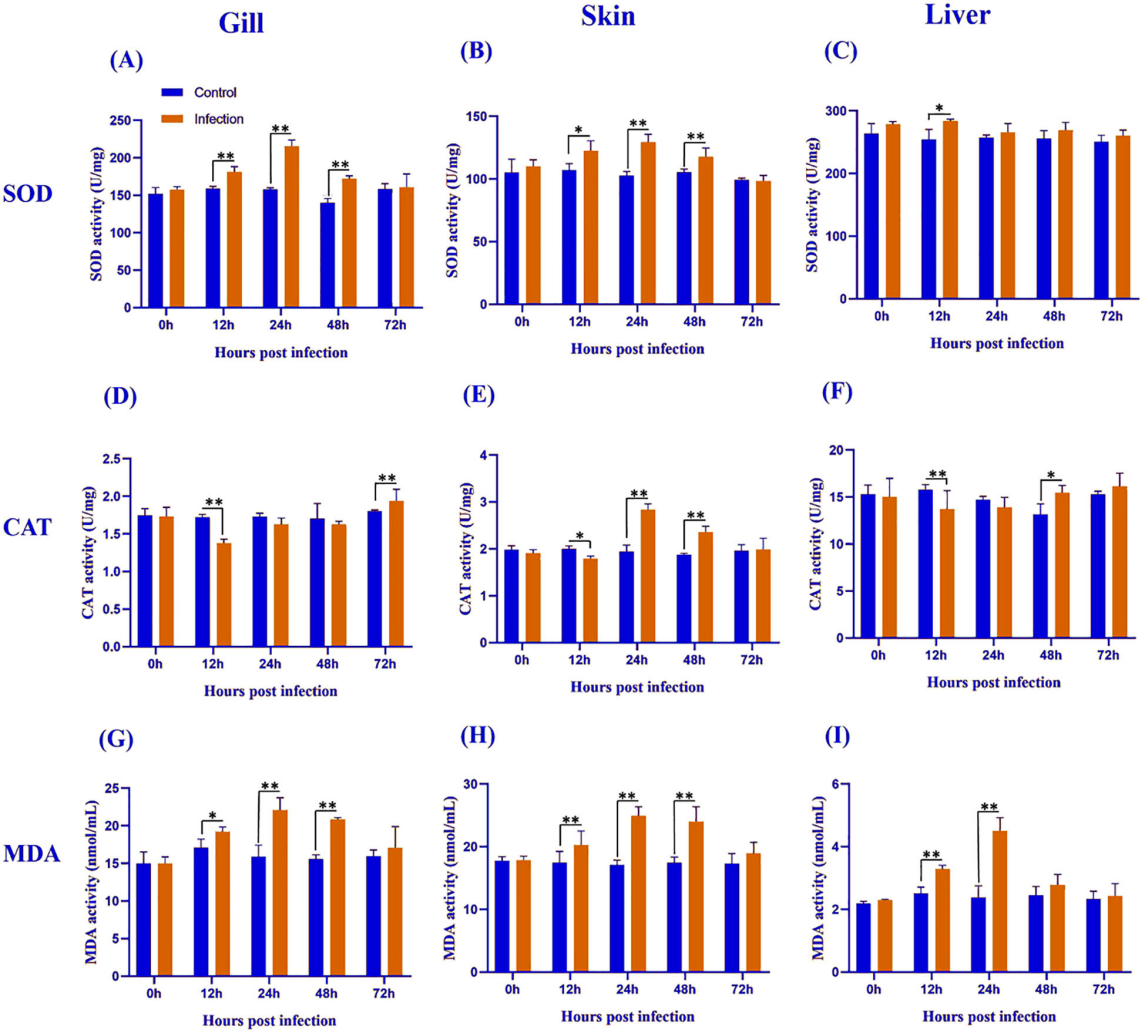
Antioxidant enzyme activity and lipid peroxidation levels in the gill **(A, D, G)**, skin **(B, E, H)**, and liver **(C, F, I)** of *L. crocea* following *Metanophrys* sp. infection. Superoxide dismutase (SOD), catalase (CAT), and malondialdehyde (MDA) levels were measured at 0, 12, 24, 48, and 72 hpi. Data are presented as mean ± SD (n=9). Asterisks indicate statistically significant differences between infected and control groups at each time point (*p<0.05, **p<0.01, Student’s test).

Superoxide dismutase (SOD) activity exhibited tissue- and time-specific responses following *Metanophrys* sp. infection. In the gill (**Figure 2A**), SOD activity was significantly elevated in the infected group at 12, 24, and 48 hpi compared to the control (*p* < 0.01), with no significant differences observed at 0 and 72 hpi (*p*>0.05). In the skin (**Figure 2B**), a moderate increase in SOD activity was detected at 12 hpi (*p<*0.05), followed by highly significant differences at 24 and 48 hpi (*p<*0.01), which returned to baseline levels by 72 hpi (*p>*0.05). Hepatic tissue (**Figure 2C**) showed a distinct pattern, with a transient but significant increase in SOD activity only at 12 hpi (*p<*0.05), and no significant differences at later time points. Collectively, these findings indicate a biphasic SOD response across all examined tissues, marked by early activation and subsequent normalization.

### 3.3 Effect of *Metanophrys* sp. infection on CAT activity in *L. crocea*

Catalase (CAT) activity varied across tissues and time points following *Metanophrys* sp. infection. In the gill (**Figure 2D**), CAT activity decreased significantly at 12 hpi and significantly increased at 72 hpi (both *p<*0.01), with no significant changes at 0, 24, or 48 hpi. In the skin (**Figure 2E**), CAT activity began to diverge at 12 hpi (*p<*0.05), reaching peak levels at 24 and 48 hpi (*p<*0.01), before returning to baseline by 72 hpi. In the liver (**Figure 2F**), CAT activity showed an inverse trend, with a marked reduction at 12 hpi (*p<*0.01) and a moderate increase at 48 hpi (*p<*0.05). These results reflect a tightly regulated tissue-specific redox response.

### 3.4 Effect of *Metanophrys* sp. infection on MDA content in *L. crocea*

Malondialdehyde (MDA), a biomarker of lipid peroxidation, was significantly elevated in all examined tissues following *Metanophrys* sp. infection. In the gill (**Figure 2G**), MDA levels increased significantly from 12 to 48 hpi (*p<*0.05), with the highest increases recorded at 24 and 48 hpi (*p<*0.01). The skin (**Figure 2H**) mirrored the gill trend, with highly significant increases throughout 12–48 hpi (*p<*0.01). In hepatic tissue (**Figure 2I**), MDA increases were transient and restricted to 12 and 24 hpi (*p<*0.01). These results suggest a strong but time-limited oxidative stress response to infection, particularly at mucosal surfaces.

### 3.5 Effect of *Metanophrys* sp. infection on LZM activity in *L. crocea*

**Figure 3:**
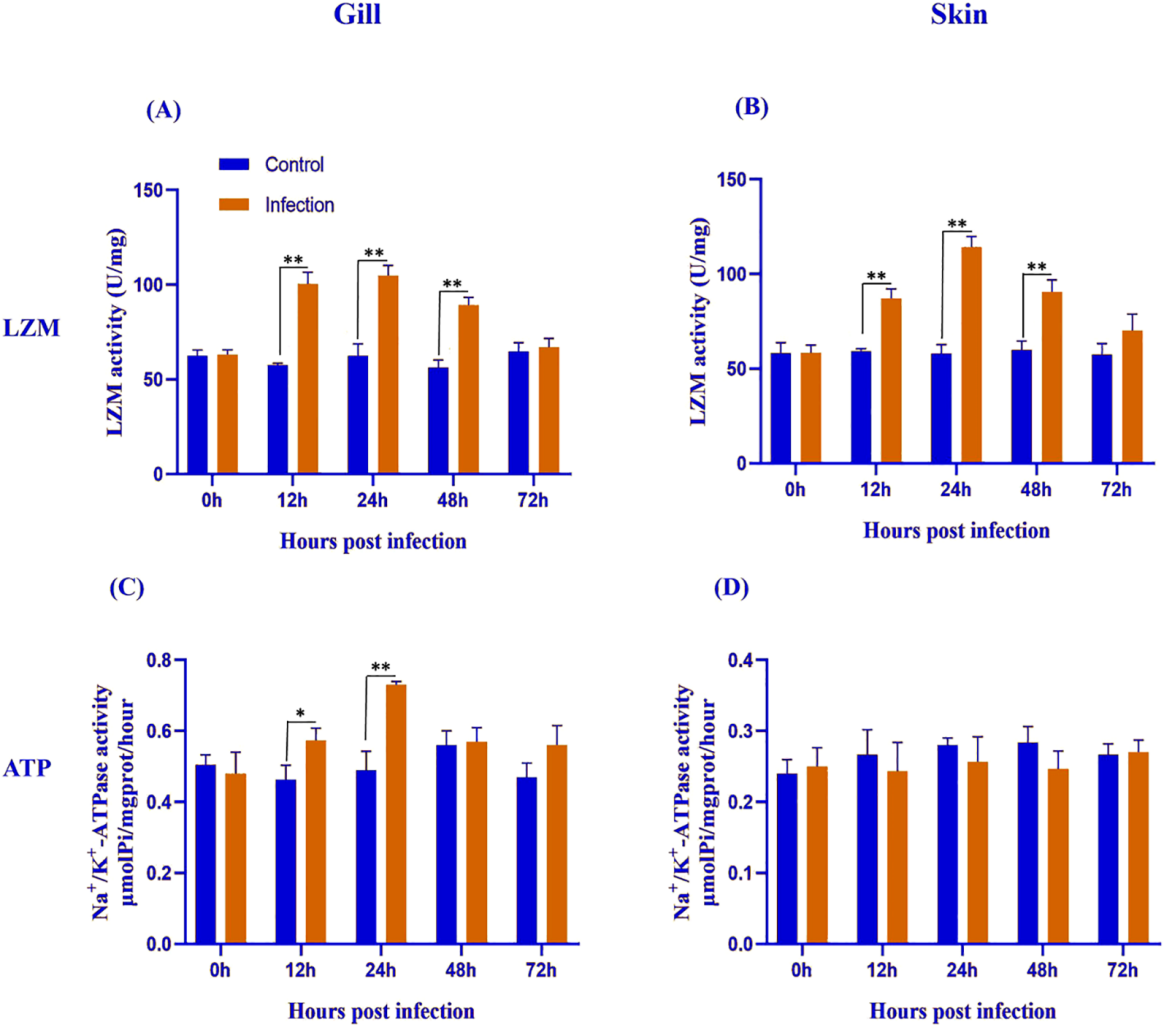
Lysozyme (LYZ) and Na^+^/K^+^-ATPase activities in the gill **(A, C)** and skin **(B, D)** of *L. crocea* following *Metanophrys* sp. infection. LZM and Na^+^/K^+^-ATPase activities were measured at 0, 12, 24, 48, and 72 hpi in both infected and control group. Values are presented as mean ± SD (n=9). Asterisks indicate statistically significant differences between infected and control samples at the same time point (*p<0.05, ** *p*<0.01, Student’s test).

Lysozyme (LZM) activity increased significantly in both gill and skin during *Metanophrys* sp. infection. In the gill and skin (**Figures 3A, B**), infected fish showed significantly elevated LZM across 12–48 hpi (*p<*0.01), but not at 0 or 72 hpi (*p>*0.05). These findings indicate rapid and transient upregulation of non-specific antimicrobial defenses at epithelial barriers.

### 3.6 Effect of *Metanophrys* sp. infection on Na^+^/K^+^- ATPase activity in *L. crocea*

Na^+^/K^+^- ATPase activity showed a tissue-specific pattern in response to *Metanophrys* sp. infection. In the gill (**Figure 3C**), ATPase activity was significantly elevated at 12 and 24 hpi (*p<*0.05), with no differences at 0, 48, or 72 hpi (*p>*0.05). In contrast, no significant differences were observed in the skin at any time point (**Figure 3D**; *p>*0.05). These results suggest that osmoregulatory adjustments to *Metanophrys* sp. infection are primarily localized to the gills.

### 3.7 RNA sequencing analysis

**Figure 4:**
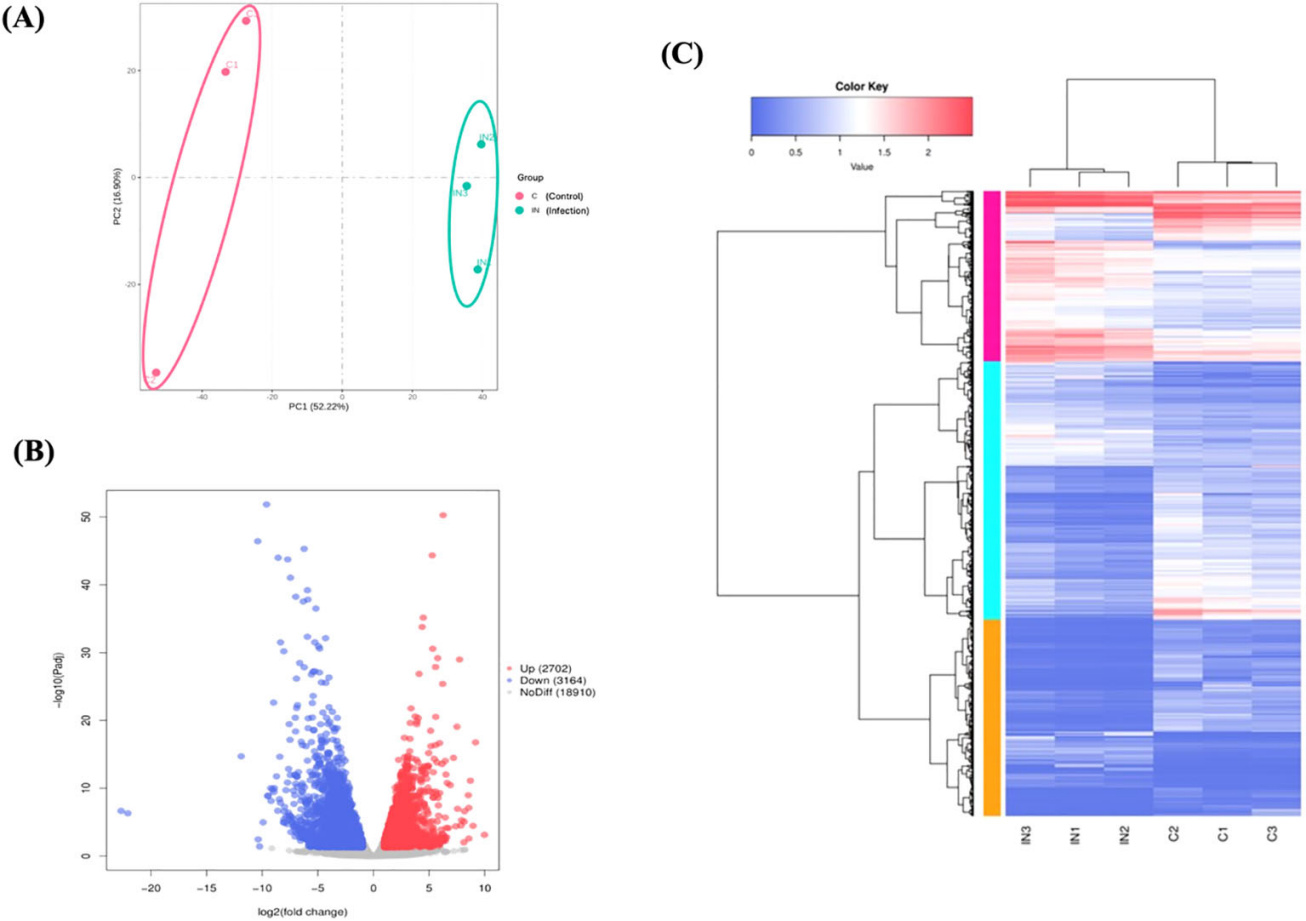
Overview of transcriptomic differences between control and infected *L. crocea* skin at 72 hpi following *Metanophrys* sp. infection. **(A)** Principal component analysis (PCA) plot showing a clear separation between control and infected samples based on global gene expression profiles. **(B)** Volcano plot of differentially expressed genes (DEGs). Red dots represent significantly upregulated genes (log_2_FC ≤ –1, adjusted *p* < 0.05). **(C)** Hierarchical clustering heatmap of DEGs. Rows represent genes and columns represent individual samples. Color intensity highlight the expression level (log_2_-transformed), with red and blue indicating up and downregulation, respectively.

A total of 151,094,218 raw reads were generated from the skin samples of both control and *Metanophrys* sp. infected groups, with each library yielding between 19 and 26 million reads. After adapter trimming and quality filtering, 134,905,552 clean reads were retained, of which 120,379,992 reads were successfully mapped to the reference genome of *L. crocea*, resulting in an overall mapping rate of 89.4% (**Table 1**). Principal component analysis (PCA) was conducted to assess the overall transcriptomic variance between control and infected groups. The PCA revealed clear separation along the first principal component (PC1), which accounted for 52,2% of the total variance in gene expression (**Figure 4A**). All biological replicates within each group clustered tightly, while control and infected samples formed distinct, non-overlapping groups, indicating that *Metanophrys* sp. infection induces broad transcriptional reprogramming in the skin of *L. crocea*.

**Table 1:** Summary of RNA-seq sample quality metrics.

Sample pairs	Clean reads	Total mapped	Clean base (bp)	Q20 (%)	Q30 (%)	GC (%)
C1	22,251,954	20,238,789 (91.60%)	6,675,586,200	99.05	96.45	48.61
C2	24,089,843	18,591,661 (88.27%)	7,226,952,900	99.24	97.11	49.93
C3	18,172,634	14,452,550 (89.39%)	5,451,790,200	99.24	97.10	50.07
IN1	24,159,117	19,277,795 (87.79%)	7,247,735,100	99.23	97.16	50.47
IN2	22,946,147	19,092,526 (88.40%)	6,883,844,100	99.25	97.14	51.27
IN3	23,285,857	17,819,754 (90.78%)	6,985,757,100	99.02	96.33	49.07

### 3.8 Differential gene expression profiling

The transcriptomic analysis of skin tissue at 72 hpi revealed extensive gene expression remodeling in *L. crocea* following *Metanophrys* sp. challenge (**Figure 4B**). A total of 6,360 differentially expressed genes (DEGs) were identified using a threshold of |log2 fold change| ≥ 1, and a Benjamini-Hochberg adjusted p-value ≤ 0.05. Among these, 3,164 genes were significantly upregulated and 2,702 were downregulated. The volcano plot graphically illustrates the broad transcriptomic shift, with significantly altered genes distributed across both upregulated (red) and downregulated (blue) categories. Most transcripts (n=18,910), shown in grey, remained unchanged, indicating a focused host response rather than a global transcriptomic dysregulation.

Hierarchical clustering of DEGs further demonstrated a clear spread between control and infected groups, with strong intra-group consistency (**Figure 4C**). The upper quadrant of the heatmap displayed genes consistently upregulated in infected samples, while the lower quadrant contained genes strongly downregulated upon infection. This transcriptional profile was consistent with the activation of canonical innate immune responses, including cytokine signaling (e.g., *ilib, il6*, *irf1*, *tgfb*), parasite recognition (e.g., *tlr5, hsp70*, *asgr2*), and inflammatory mediatiors (e.g., *cxcl8, mmp9, ptgs2*). Genes involved in epithelial signaling (e.g., *cxcr2*, *etvs5*) and metabolic regulation (*tshib*, *camp*, *pnpla2*, *irs2*) were also differentially expressed (**Supplementary Table 2**).

Importantly, several of the differentially upregulated genes showed dual functional annotation in both immune and metabolic pathways, highlighting a biologically significant interphase between these systems. Genes such as *il1b*, *il6*, *cxcl8*, *mmp9*, and *pnpla2* were simultaneously mapped to immune related KEGG pathways (e.g., *NFkB* signaling and *TNF* signaling) and metabolic processes (e.g., *FoxO signaling*, *cellular senescence*) (**Supplementary Table 3**). Protein-level annotation using the Swiss-Prot database further validated their roles in chemotaxis, lipid remodeling, extracellular matrix degradation, and cytokine signaling (**Supplementary Table 4**). This intersection indicates that *L. crocea* mounts a highly coordinated immunometabolic response to constraint the parasite spread while preserving tissue integrity.

Conversely, multiple downregulated genes were associated with cellular homeostasis (e.g., *aqp3*, *fgfr2*), ion transport (e.g., *slc9a6*, *atp18*), and structural barrier function (e.g., *mmp3*, *cldn*), reflecting a prioritization of cellular resources during immune engagement (**Supplementary Table 5**). Together, these results demonstrate that *Metanophrys* sp. infection triggers a robust and integrated transcriptomic program in *L. crocea*, characterized by the simultaneous immune activation and metabolic adaptation in the skin.

### 3.9 Gene ontology enrichment analysis

**Figure 5:**
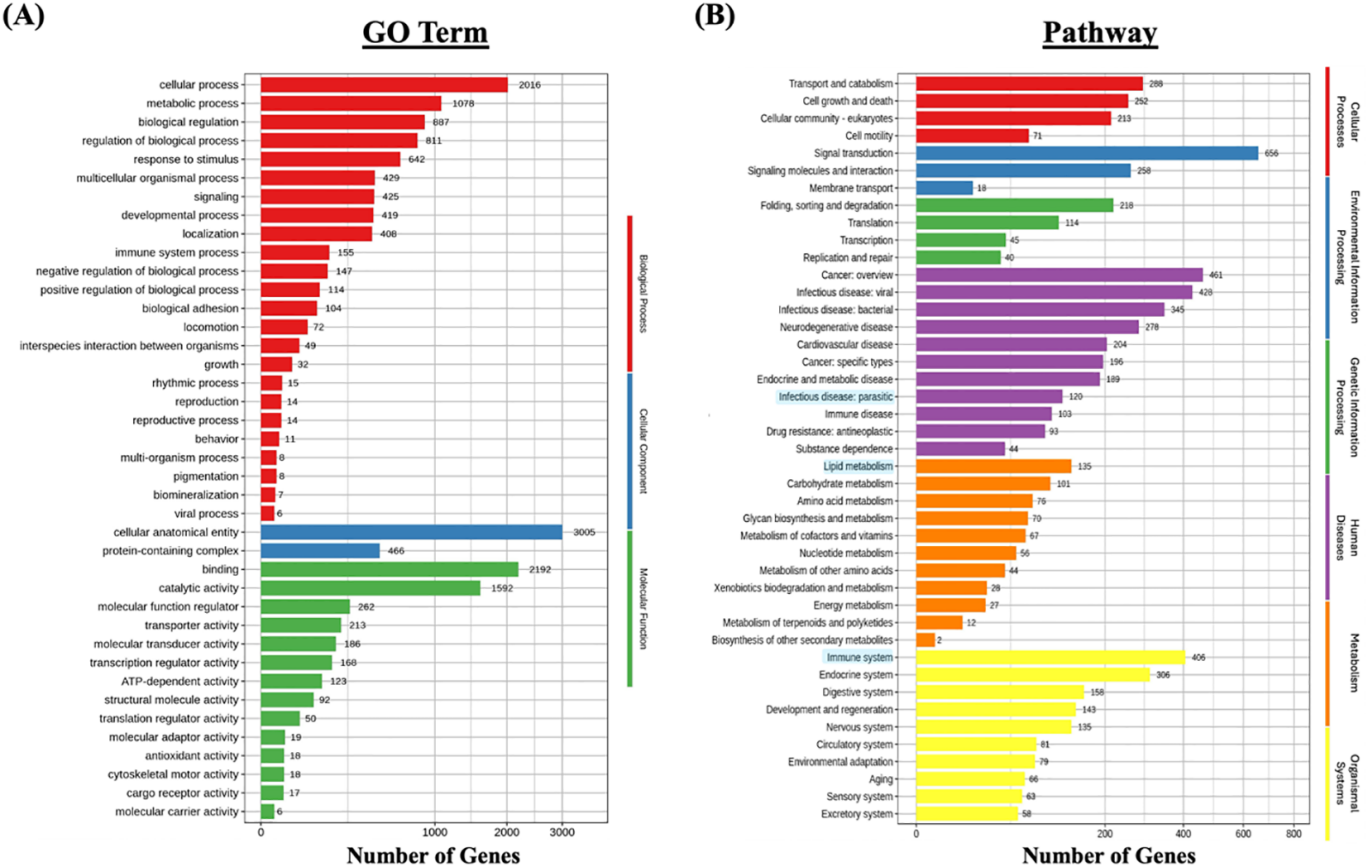
Functional enrichment analysis of differentially expressed genes (DEGs) in *L. crocea* skin following *Metanophrys* sp. infection. **(A)** Gene Ontology (GO) classification of DEGs based on biological process (red), cellular component (blue), and molecular function (green). Bars represent the number of DEGs annotated within each GO term. **(B)** KEGG pathway enrichment analysis. DEGs were grouped under major KEGG categories, including metabolism (orange), environmental information processing (green), genetic information processing (purple), cellular processes (blue), and organismal systems (yellow). Bars indicate the number of DEGs associated with each pathway.

GO enrichment analysis was performed to classify the functional roles of the DEGs in response to *Metanophrys* sp. infection. Significant enrichment was observed across all three primary GO categories: biological processes, cellular component, and molecular function (**Figure 5A**). Within the biological process category, the most enriched terms included *cellular process* (n=2016), *metabolic process* (n=1078), and *biological regulation* (n=887) indicating extensive physiological reprogramming in infected tissues. In the cellular component category, *cellular anatomical entity* (n=3,005) and *protein-containing complex* (n=466) were most represented, highlighting significant shifts in subcellular localization and macromolecular organization. For molecular function, enriched terms included *binding* (n=2192), *catalytic activity* (n=1,592), and *molecular function regulator* (n=262), suggesting enhanced engagement of enzymatic and regulatory pathways. Moreover, in support of the immunometabolic signature, enriched metabolic term under the biological process and molecular function categories included *oxidoreductase activity*, *cofactor binding*, and *ATP-dependent activity*, which are indicative of mitochondrial involvement and energy redistribution. Together, these findings underscore the activation of diverse cellular, structural, immune, and metabolic programs in the skin of *L. crocea* as part of a complex response activated upon parasite invasion.

### 3.10 KEGG pathway analysis

To further investigate the biological pathways affected by infection, KEGG pathway enrichment analysis was conducted (**Figure 5B**). A total of 120 DEGs were associated with parasitic infections, while 406 genes were mapped to immune system-related pathways within the broader organismal systems category. Notably, pathways such as *Signal transduction* (n=656), *Transport and catabolism* (n=288), and *Signaling molecules and interaction* (n=258) were significantly enriched, confirming the activation of canonical immune pathways.

In parallel, metabolic adaptations were evidenced by the enrichment of energy-associated pathways. Specifically, *Lipid metabolism* (n=135), *Carbohidrate metabolism* (n=101), and *Amino acid metabolism* (n=75) were among the most represented metabolic categories, indicating enhanced mitochondrial engagement and glucose utilization during the host response. These findings reinforce the existence of an immunometabolic interface, wherein metabolic resources are mobilized in concert with immune signaling to sustain tissue-level defense and parasite control.

**Figure 6:**
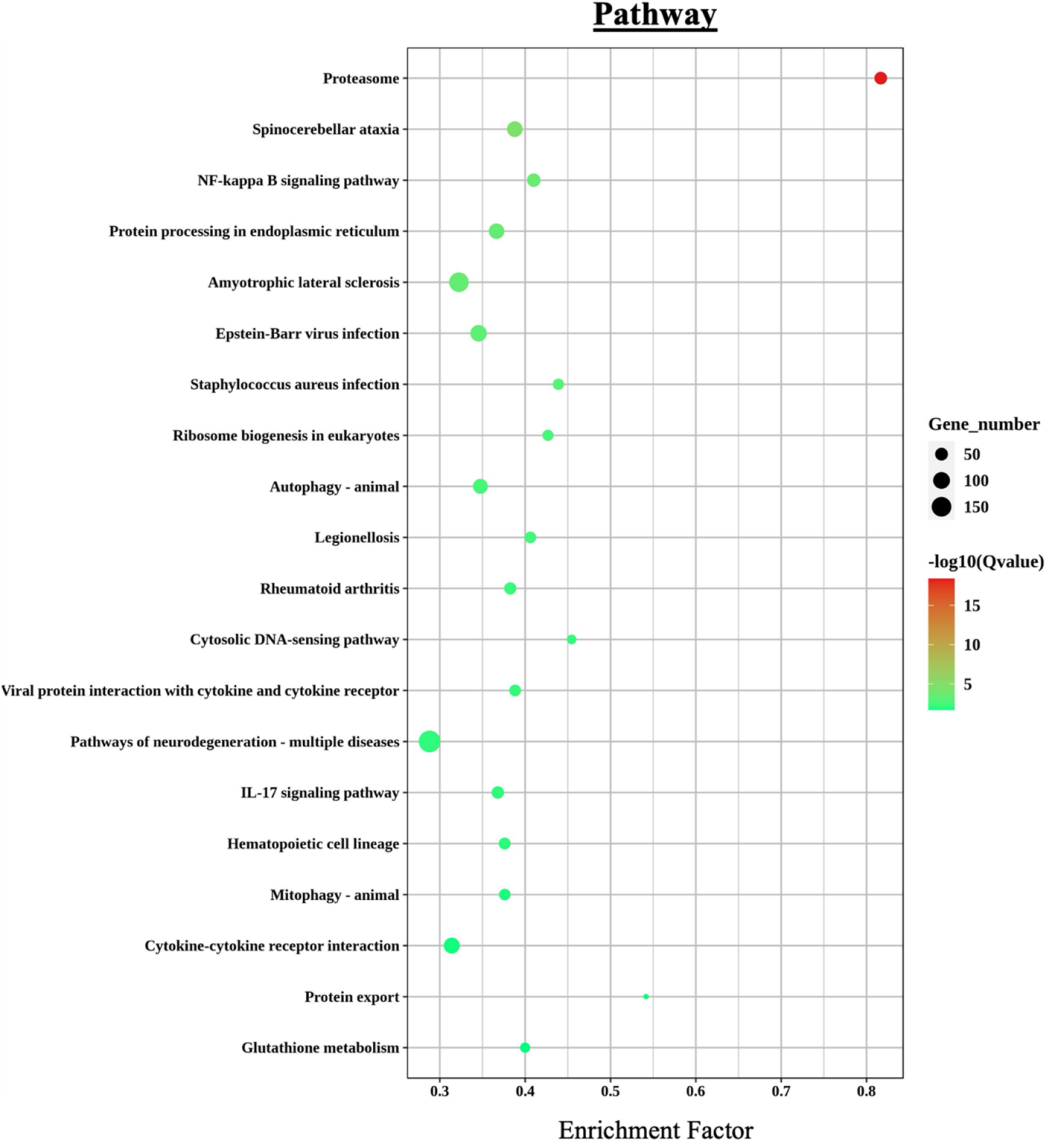
KEGG pathway enrichment analysis of differentially expressed genes (DEGs) in *L. crocea* skin following *Metanophrys* sp. infection. The bubble plot displays the top enriched pathways based on enrichment factor and gene count. Bubble size corresponds to the number of genes annotated to each pathway, while color intensity reflects statistical significance –log_10_(Q-value). Notable pathways include *proteasome*, *NF-kappa B signaling*, *IL-17 signaling*, and *cytokine–cytokine receptor interaction*, which are central to immune regulation and stress responses.

Additional enriched KEGG categories included *signal transduction*, *infectious diseases*, and *cellular processes*. A bubble chart of the top 20 immunometabolic and infection-related KEGG terms (**Figure 6**) revealed a particular high enrichment score (–log10 p-value) for *proteasome*, *NF-kappa B signaling pathway*, and *protein processing in the endoplasmic reticulum*, among several others.

### 3.11 Immune-related pathway analysis

An integrated GO/KEGG analysis identified 28 immunometabolic-related and 6 parasitic disease-related pathways significantly enriched in the skin transcriptome of *L. crocea* following *Metanophrys* sp. infection (**Tables 2**, **3**). Among the immune-related pathways, the most prominently enriched were *chemokine signaling* (71 genes), *NOD-like receptor signaling* (61 genes), *Th17 differentiation* (49 genes), and *Platelet activation* (n=49). These were followed in magnitude by *IL-17 signaling* (46 genes), and *Fc gamma R-mediated phagocytosis* (45 genes), among many other pathways and genes highlighting robust activation of innate and adaptive immune axes.

**Table 2:** KEGG pathways significantly enriched in immune and metabolic responses of *L. crocea* skin following *Metanophrys* sp. infection.

Pathway ID	KEGG pathway	Gene count
ko04062	Chemokine signaling pathway	71
ko04621	NOD-like receptor signaling pathway	61
ko04659	Th17 cell differentiation	49
ko04611	Platelet activation	49
ko04657	IL-17 signaling pathway	46
ko04625	C-type lectin receptor signaling pathway	45
ko04666	Fc gamma R-mediated phagocytosis	45
ko04670	Leukocyte transendothelial migration	43
ko04640	Hematopoietic cell lineage	41
ko04658	Th1 and Th2 cell differentiation	41
ko04660	T cell receptor signaling pathway	40
ko04613	Neutrophil extracellular trap formation	36
ko04662	B cell receptor signaling pathway	32
ko04620	Toll-like receptor signaling pathway	32
ko04650	Natural killer cell mediated cytotoxicity	32
**ko05417**	Lipid and arteriosclerosis	31
ko04612	Antigen processing and presentation	31
ko04060	Cytokine-cytokine receptor interaction	29
ko04623	Cytosolic DNA-sensing pathway	25
ko04610	Complement and coagulation cascades	25
ko04022	RIG-I-like receptor signaling pathway	24
**ko05231**	*Choline metabolism*	18
ko04664	Fc epsilon RI signaling pathway	18
**ko00561**	*Glycerolipid metabolism*	17
**ko00564**	*Glycerophospholipid metabolism*	17
**ko00190**	*Oxidative phosphorylation*	15
ko04624	Toll and Imd signaling pathway	14
**ko00010**	*Glycolisis/Gluconeogenesis*	13

Bold IDs denote core metabolic pathways.

**Table 3:** KEGG pathways significantly enriched in *L. crocea* skin associated with parasitic diseases following *Metanophrys* sp. infection.

Pathway ID	KEGG pathway	Gene count
ko05145	Toxoplasmosis	50
ko05142	Chagas disease	44
ko05146	Amoebiasis	43
ko05140	Leishmaniasis	29
ko05144	Malaria	26
ko05143	African trypanosomiasis	13

Notably, several metabolic pathways were also significantly represented, including *Lipid and arteriosclerosis* (31 genes), *Choline metabolism* (18 genes), *Glycerolipid metabolism* (17 genes), and *Oxidative phosphorylation* (15 genes) among others. This enrichment supports a coordinated metabolic reprogramming concurrent with immune activation, indicative of a functional immunometabolic interface.

Transcriptomic analysis revealed that multiple DEGs were implicated in pathogen recognition, downstream immune signaling, and immunometabolic pathways (**Table 4**). Notably, genes such as *foxo1*, *raf1*, *rohaa*, *hsp90*, *il6*, and *akt1* were significantly upregulated, while *src*, *fadd*, *p38*, *plc*, and *pka* were significantly downregulated. These genes are central to chemokine signaling, NOD-like and Toll-like receptor pathways, and cytokine and MAPK signaling modules—indicating broad transcriptional activation of immune responses and concurrent modulation of stress and apoptosis-related pathways.

**Table 4:** Representative immune-related pathways and associated genes showing differential expression in the skin of *L. crocea* following *Metanophrys* sp. infection.

Pathway	Gene abbreviation	Gene name	Fold change
Chemokine signaling	*aktl*	Protein kinase B	1.9129
	*foxol*	Forkhead box O	1.5408
	*rafl*	Rapidly accelerated fibrosarcomal	1.4816
	*roha*	Ras homolog gene family, member A	1.3797
	*pka*	Protein kinase A	-1.2162
	*src*	Src protein	-1.8591
	*plc*	Phospholipase C	-1.9291
NOD-like receptor signaling	*il6*	Interleukin-6	5.8994
	*trx*	Thioredoxin	2.3240
	*hsp90*	Heat Shock Proteins 90	1.8613
	*tbkl*	TANK-binding kinase	1.8442
	*sgtl*	Protein SGT1 homolog isoform X2	1.5898
Toll-like receptor signaling	*lbp*	Bactericidal permeability-increasing protein	-1.2233
	*fadd*	FAS-associated death domain protein	-2.3900
Th17 cell differentiation	*hif-1*	Hypoxia-inducible factor 1-alpha	2.2347
	*p38*	p38 MAPK	-1.0862
	*mhcii*	MHC class II protein complex	-1.7432
	*cd3*	T-cell surface glycoprotein CD3 epsilon chain	-2.6207
C-type lectin receptor signaling	*ppp3r*	Calcineurin subunit B type 1-like	1.2822
	*p38*	Mitogen-activated protein kinase	-1.0862
	*casp8*	Caspase-8-like	-2.1220
B cell receptor signaling	*nfatc3*	Nuclear factor of activated T-cells, cytoplasmic 3	1.6369
	*malt]*	Mucosa-associated lymphoid tissue lymphoma translocation protein 1	-1.3903
	*nfatc2*	Nuclear factor of activated T-cells, cytoplasmic 2	-2.7276

Gene regulation is indicated as upregulated (green) or downregulated (red) based on RNA-seq analysis. Listed genes are involved in key immune downstream signaling.

**Figure 7:**
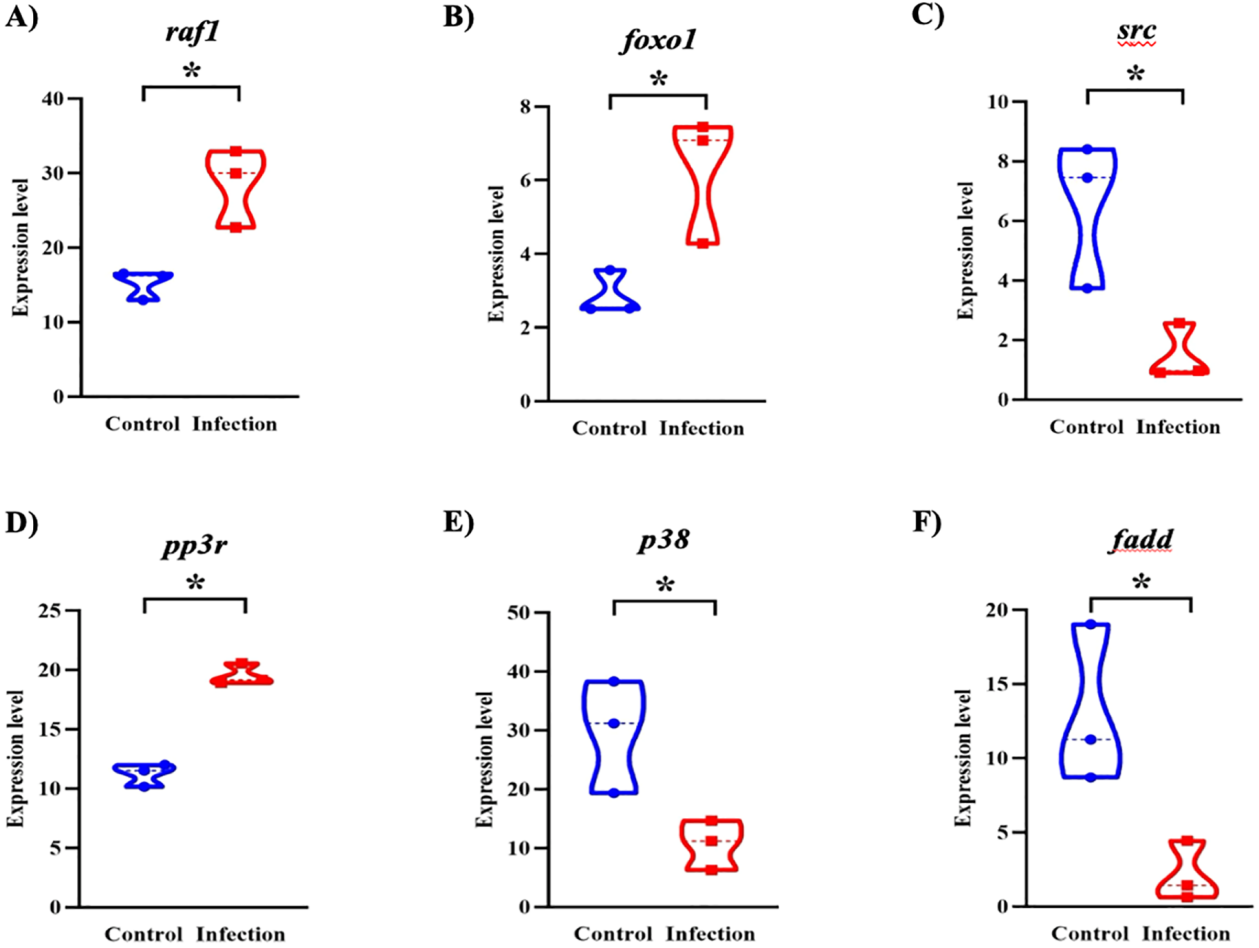
Violin plots showing differential expression of key immune-related genes in the skin of *L. crocea* following *Metanophrys* sp. infection. Gene expression levels of **(A)** *raf1*, **(B)** *foxo1*, **(C)** *src*, **(D)** *ppp3r*, **(E)** *p38*, and **(F)** *fadd* were compared between control and infected groups. Asterisks denote statistically significant differences (*p* < 0.05) based on unpaired Student’s *t*-test.

These expression patterns were further visualized through targeted analysis of six representative genes. As shown in the violin plots (**Figure 7**), infected fish exhibited significantly higher expression of *raf1*, *foxo1*, and *ppp3r* (p < 0.05), supporting enhanced activation of chemokine signaling, metabolic regulation, and T cell modulatory mechanisms. In particular, the upregulation of *foxo1* and *ppp3r*, genes linked to energy metabolism and calcium-dependent T cell activation, highlights the role of immunometabolic reprogramming during host response. In contrast, *src*, *p38*, and *fadd* were significantly downregulated (p < 0.05), suggesting a targeted dampening of specific kinase signaling and apoptotic pathways, potentially to preserve epithelial integrity and sustain local immune cell function under infection-induced stress. These findings support the concept that *L. crocea* mounts a coordinated immunometabolic response to *Metanophrys* sp., integrating pro-inflammatory signaling with metabolic adaptation at the mucosal surface.

**Figure 8:**
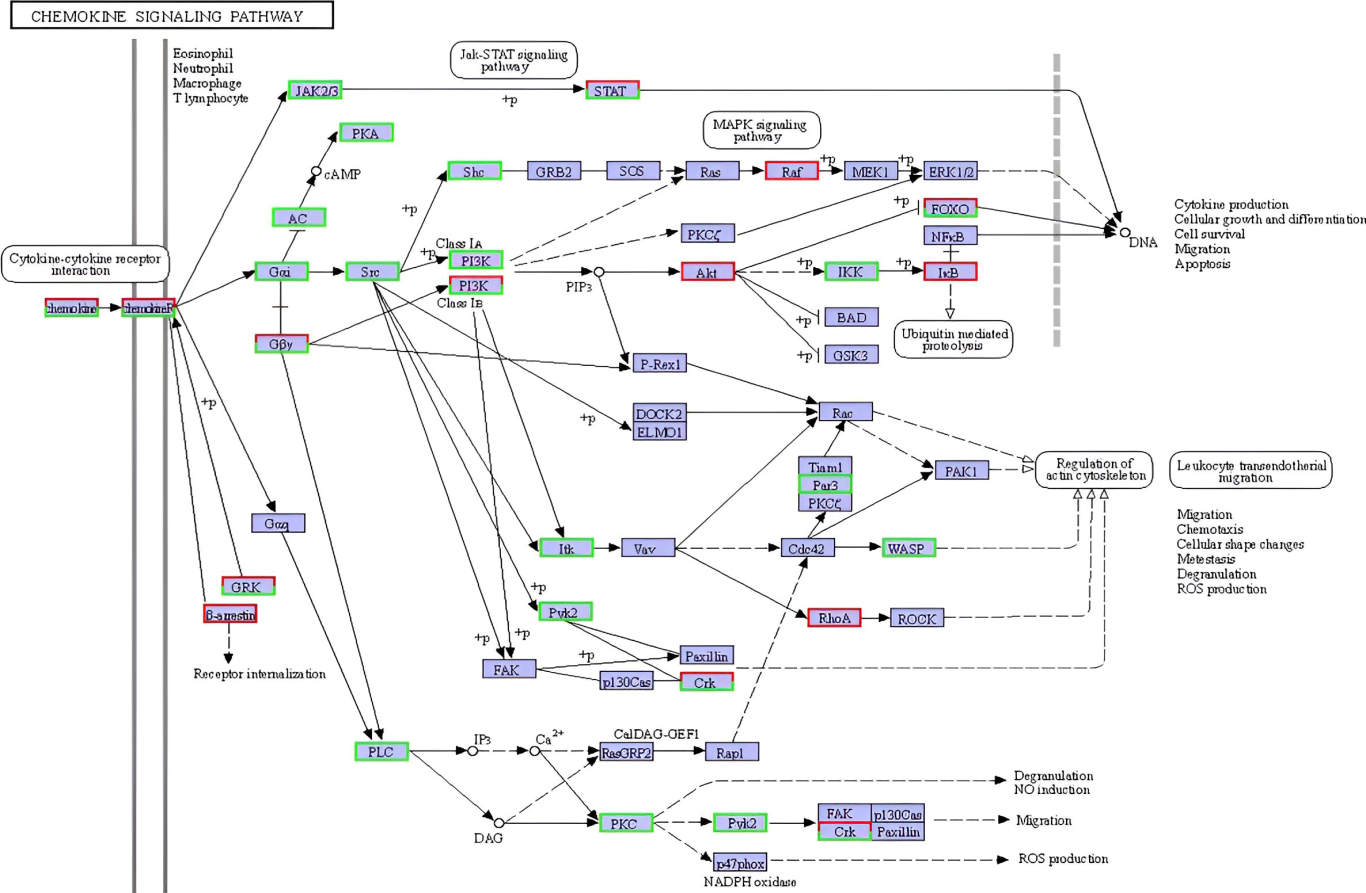
Reference KEGG pathway map of the cytokine–cytokine receptor interaction signaling pathway (ko04060) enriched in *L. crocea* skin following *Metanophrys* sp. infection. Differentially expressed genes (DEGs) are mapped onto the pathway. Boxes in green indicate upregulated genes, while red indicates downregulated genes. This pathway illustrates the activation of downstream immune responses, including JAK–STAT, MAPK, PI3K–Akt, and PLC signaling cascades, contributing to leukocyte recruitment, cytokine production, and inflammation.

Additionally, the KEGG chemokine signaling pathway map (**Figure 8**) further illustrates the complex regulation of downstream effectors, with a subset of genes exhibiting pathway-specific modulation patterns, some upregulated to facilitate leukocyte activation and chemotaxis, and others downregulated, potentially to control the inflammatory process triggered during the parasite infection process. Collectively, these enriched pathways reflect a highly coordinated and multifaceted transcriptional immune response of *L. crocea* skin to *Metanophrys* sp. infection.

### 3.12 Transcriptomic validation by qPCR

**Figure 9:**
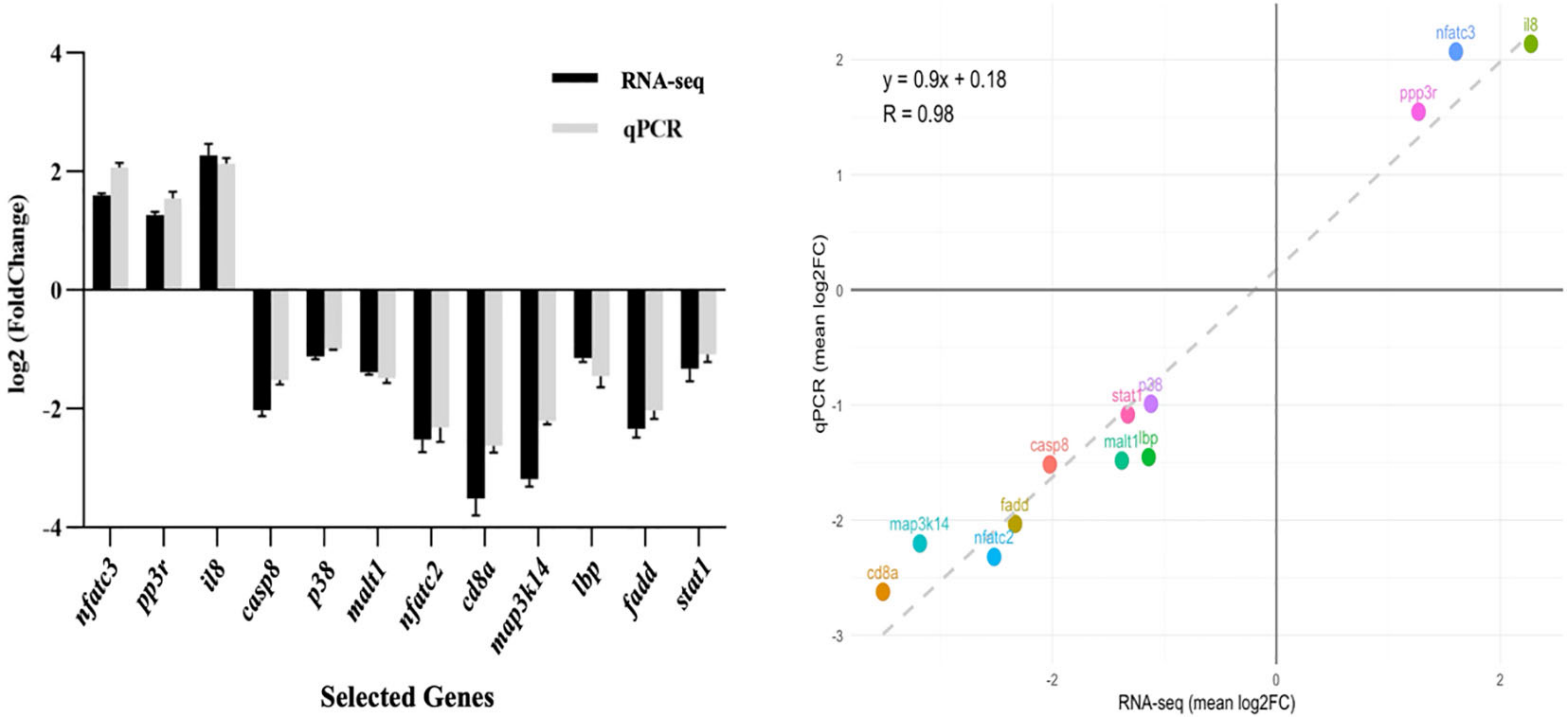
Validation of RNA-seq data by qPCR. (Left panel) Comparison of log_2_ fold change (FC) values for 12 selected genes involved in immune and metabolic pathways as determined by RNA-seq (black bars) and confirmed by qPCR (grey bars). Expression trends are consistent between both methods. (Right panel) Correlation analysis between RNA-seq and qPCR datasets. The scatter plot shows a strong positive correlation (*R²* = 0.98), confirming the reliability of the transcriptomic data.

qPCR validation of 12 randomly selected immune-related genes demonstrated strong concordance with RNA-seq data (**Figure 9**), confirming methodological reliability. The expression patterns showed remarkable consistency between both analytical approaches, validating our transcriptomic findings.

## 4 Discussion

This study provides the first integrated enzymatic-transcriptomic analysis of *L. crocea* during infection with *Metanophrys* sp., offering a systems-level view of mucosal immune activation and metabolic reprogramming. Our findings reveal a temporally coordinated host response that aligns with canonical vertebrate immune mechanisms, while exhibiting teleost-specific adaptations, particularly at the skin barrier.

The adaptive surface mechanics of scuticociliates, notably, cilia-driven propulsion and rapid morphological plasticity, can hinder neutrophil access and impair phagocytic efficiency (33). In our study, following *Metanophrys* sp. exposure, antioxidant enzymes SOD and CAT were significantly upregulated in the gill and skin between 12–24 hpi (*p<*0.05), returning to baseline by 72 hpi. This biphasic pattern suggests that SOD and CAT function as front-line defenses, mitigating reactive oxygen species (ROS), such as superoxide and hydrogen peroxide during early invasion. Similar kinetics were reported in turbot (*Scophthalmus maximus*) infected with *Philasterides dicentrarchi*, where early-phase ROS production triggered antioxidant responses (25, 34). However, in the turbot model, antioxidant activation alone was insufficient for parasite clearance, likely due to parasite-mediated immune evasion strategies (35). Our findings, together with comparable reports in *Mesanophrys*-infected mud crabs (36), underscore the conserved role for enzymatic redox buffering in host protection across marine invertebrates and teleosts.

Concurrently, MDA, a marker of lipid peroxidation, was significantly elevated (*p <* 0.01) between 12–24 hpi, indicating oxidative membrane damage as a hallmark of early pathophysiological responses. Although MDA is traditionally used as an indicator of oxidative stress (37), transient MDA accumulation under regulated redox conditions may also act as a signaling intermediate, promoting the transcription of cytoprotective and immunomodulatory genes. This dual role of MDA has been also observed in Nile tilapia (*Oreochromis niloticus*), where elevated MDA levels during *Clinostomus* infection correlated with increased expression of *il1b*, *mhci*, and *mhcii* suggesting a redox-sensitive immune signaling role (38). Accordingly, our data indicate that *L. crocea* harnesses this redox-mediated mechanism to initiate early immune responses and maintain inflammatory control during *Metanophrys* sp. infection.

Interestingly, despite the slight but significant redox imbalance observed in the liver, suggesting systemic generation of reactive oxygen species (ROS) in response to *Metanophrys* sp. infection, our previous findings (11), supported by the current study’s results, indicate that the primary redox disruptions remain localized to mucosal surfaces such as the skin, gills, and fins, with no detectable parasite DNA in internal organs like the liver or brain. Accordingly, this study concentrates on direct mucosal responses, while the investigation of systemic downstream effects is reserved for future research.

Consistent with this localized response, upregulation of lysozyme and Na^+^/K^+^-ATPase activity in mucosal tissues of *L. crocea* indicates the presence of localized epithelial stress and activation of humoral immune defenses at mucosal barrier surfaces. Elevated LZM levels in both gill and skin align with finding obtained in *Takifugu rubripes* following *Uronema marinum* vaccination (20), supporting its role as a conserved mucosal immune marker in teleosts. In contrast, Na^+^/K^+^-ATPase activity was significantly elevated only in the gill, suggesting a tissue-specific osmoregulatory response to parasitic infection. This gill specific induction mirrors previous findings in *Tetraodon nigroviridis* exposed to epithelial damage (21), indicating that *L. crocea* relies on targeted ion transport regulation in the gill to preserve osmotic balance during infection. These compartmentalized physiological responses are further supported by transcriptional profiling, as shown by RNA-seq analysis of infected skin as described below.

To further dissect *L. crocea* immune response mechanisms to *Metanophrys* sp. infection, RNA-seq analysis was performed on infected skin at 72 hpi. A total of 3,164 genes were upregulated and 2,702 downregulated, indicating substantial transcriptional remodeling. Enrichment analysis identified the activation of key immune signaling pathways, including NOD-like receptor, IL-17, and chemokine signaling. These findings are consistent with previous transcriptomic studies in *L. crocea* infected with *Cryptocaryon irritans*, which reported progressive activation of proinflammatory and stress-related genes in the skin (39). The NOD-like receptor pathway is critical for sensing intracellular pathogens, fighting parasitic infections, and initiating inflammatory cascades (40). Our results support the hypothesis that *Metanophrys* sp. is recognized by pattern recognition receptors (PRRs) targeting parasite-derived RNA, DNA, and glycoproteins (41–43), leading to activation of VEGF, TNF, and MAPK signaling pathways, which coordinate leukocyte recruitment, vascular integrity, and stress responses (44).

Transcriptomic remodeling further revealed that multiple canonical immune pathways were modulated in a manner indicative of immune-metabolic coordination. For instance, the upregulation of *raf1* and *foxo1* within the chemokine signaling cascade highlights the likely integration of immune cell trafficking with metabolic regulation. The observed transcriptional increase of these genes may reflect a concerted mobilization of immunometabolic circuits during early mucosal defense. Specifically, *foxo1* encodes a key forkhead box transcription factor critical for orchestrating cellular adaptation to stress; it modulates oxidative stress responses by upregulating antioxidant genes such as *sod2* and *cat* (45), regulates glucose metabolism via insulin signaling and gluconeogenesis (46), and mediates anti-inflammatory responses by suppressing *NfkB*-driven cytokine expression (47). Therefore, *foxo1* induction in infected skin likely represents a strategic host response to mitigate tissue damage, preserve epithelial integrity, and maintain metabolic homeostasis under parasitic challenge. Additionally, *raf1*, a MAPKKK involved in Ras-dependent signaling (48), has been linked to thrombocyte activation and cellular survival pathways, suggesting an ancillary role in coordinating immune and metabolic cues at the infection site.

In contrast, the downregulation of *src*, a component of early kinase signaling (49), suggests a negative feedback loop aimed at preventing hyperactivation of immune responses and limiting collateral tissue damage. Furthermore, within the Th17 differentiation and Toll-like receptor (TLR) signaling pathways, the divergent regulation of the *pp3r* (upregulated) and *p38*/*fadd* (downregulated) suggests a selective engagement of pro-inflammatory and apoptotic signaling modules, skewing the response toward controlled inflammation with minimal cytotoxicity (50). This transcriptional architecture reflects a nuanced host strategy that integrates immune activation with metabolic constraints, a hallmark of immunometabolic regulation. Similar immunometabolic trade-offs have been described in mammals during protozoan infections (51), where a balanced inflammatory profile is essential to avoid tissue damage while ensuring infection clearance (52). Collectively, these pathway-specific changes provide mechanistic support for the hypothesis that *L. crocea* mounts a finely tuned mucosal immune response, leveraging immunometabolic programs to maintain epithelial function and sustain host fitness during *Metanophrys* sp. infection, extending observations made in other vertebrate models of protozoan infection.

Among adaptive immune components, Th17-associated cytokines were particularly prominent. The consistent upregulation of *il1b*, *il6*, *cxcl8*, *ptgs2*, and *mmp9* indicates activation of the IL-17 axis, a pivotal mucosal immune pathway also implicated in *Toxoplasma gondii*, *Trypanosoma carassii*, and *Philasterides* infections in teleosts (53–55). The IL-17 pathway is known to promote leukocyte activation, epithelial repair, and inflammation, which are all critical during protozoan colonization (56, 57). Notably, downstream effectors such as *cox2*, *cxcl8*, *mmp9*, and *hsp90* were also upregulated, reflecting a robust pro-inflammatory and epithelial repair response in the skin of infected fish. These genes are functionally associated with thrombocyte activation (58), implicating a role for thrombocytes in immune modulation and vascular stabilization during infection (59).

This interpretation is supported by the identification of platelet activation signaling, reflected in the upregulation of ten thrombocyte-associated genes, suggesting a coagulative response at mucosal surfaces. While traditionally associated to hemostasis, emerging evidence supports the role fish thrombocytes, the functional analogous to platelets in fish as active immune effectors (59). As previously proposed, coagulation may serve to contain parasites at infection sites, preventing systemic dissemination through blood vessels. In our study, the platelet activation in the skin likely reflects local hemorrhage and epithelial disruption, along with cytokine production and immune cells recruitment. Similar patterns were observed in *Epinephelus coioides* infected with *Vibrio alginolyticus* (60), reinforcing the concept of conserved hemostatic-immunological integration in *L. crocea* during parasitic infection.

Consistent with this, the chemokine signaling pathway was significantly upregulated, supporting its role in leukocyte chemotaxis and inflammation during parasitic infections. Previous studies reported increased chemokine receptors expression in *E. coioides* skin infected with *C. irritans* infection (61). In *L. crocea*, *Metanophrys* sp. infection led to increased expression of *raf1*, *rhoA*, *akt*, indicating activation of intracellular signaling pathways involved in inflammation and tissue remodeling. These findings highlight the intertwined roles of chemokine signaling and thrombocyte-mediated responses in orchestrating local immune defense.

Critically, this study also demonstrates a transcriptional convergence of immune signaling with metabolic regulation, supporting the growing recognition of immunometabolism as a central axis in teleost immunity (26, 62, 63). Key regulatory genes such as *akt1*, *foxo1*, and *pnpla2* bridge lipid mobilization, redox homeostasis, and glucose metabolism with immune function. This suggests that *L. crocea* mobilizes metabolic resources in parallel with inflammatory programs, in line with immunometabolic paradigms observed in mammalian macrophages and T cells (64). Contrasting with earlier teleost studies that examined these processes independently, our data demonstrate concurrent enrichment of glycolysis, lipid metabolism, and cytokine signaling, underscoring a functional interface between metabolic adaptation and immune activation.

Unlike other protozoan infections where systemic dissemination occurs, PCR data confirmed *Metanophrys* sp. remains confined to external epithelial tissues in *L. crocea*. This spatial restriction may explain the skin dominant transcriptomic signature and the lack of systemic metabolic disruption. Nonetheless, the magnitude of transcriptional remodeling underscores the skin’s role as an immunologically active barrier rather than a passive infection site (65, 66). In addition, downregulation of genes involved in epithelial integrity and water transport (*fgfr2*, *cldn*, *aqp3*) suggests parasite induced barrier compromise. Additionally, enrichment of parasite-associated proteases (*pp2a*, *pi3k*, *pkc*, *casp3*) further suggests active modulation of host immune and apoptotic pathways, consistent with evasion strategies previously described in *Uronema* and *Philasterides* infection models (67, 68).

## 5 Conclusion

This study demonstrates that *L. crocea* mounts a coordinated, multi-layered response to *Metanophrys* sp. infection, characterized by early oxidative stress regulation, activation of innate immune signaling, and integration of metabolic and inflammatory pathways. The skin functions as a dynamic immunological interface, engaging in PRR-mediated detection, metabolic adaptation, and thrombocyte-associated vascular modulation. Upregulation of chemokine signaling highlights active leukocyte recruitment and tissue repair, while enrichment of thrombocyte-related (platelet activation) pathways suggests localized coagulation as a mechanism to limit parasite dissemination. These findings support the emerging concept that immunometabolism is a conserved vertebrate defense strategy and identify candidate molecular targets for immunodiagnostics and disease management in aquaculture. However, this study is limited by its temporal resolution, capturing host responses at a single time point (72 hpi), and by its spatial focus on skin tissue alone. Future work should incorporate longitudinal sampling and multi-tissue transcriptomic profiling, coupled with functional validation of key genes and pathways, to fully elucidate the dynamics and systemic coordination of host responses during scuticociliate infection.

## Data Availability

The datasets presented in this study can be found in online repositories. The names of the repository/repositories and accession number(s) can be found in the article/**Supplementary Material**.

## References

[1] FAO. The State of World Fisheries and Aquaculture (SOFIA) 2024 – Blue Transformation in action. Rome, Italy: FAO (2024). doi: 10.4060/cd0683en

[2] Shinn AP Avenant-Oldewage A Bondad-Reantaso MG Cruz-Laufer AJ García-Vásquez A Hernández-Orts JS. A global review of problematic and pathogenic parasites of farmed tilapia. Rev Aqua. (2023) 15:92–153. doi: 10.1111/raq.12742

[3] Zhang TT Fan X Gao F Al-Farraj SA El-Serehy HA. Further analyses on the phylogeny of the subclass Scuticociliatia (Protozoa, Ciliophora) based on both nuclear and mitochondrial data. Mol Phylogenet Evol. (2019) 139:106565. doi: 10.1016/j.ympev.2019.106565, PMID: 31326515

[4] Piazzon C Leiro J Lamas J. Reprint of “Fish immunity to scuticociliate parasites. Dev Comp Immunol. (2014) 43:280–9. doi: 10.1016/j.dci.2013.11.015, PMID: 24309548

[5] Zhang S. Diagnosis and treatment of a ciliate endoparasitic in the Large Yellow Croaker (*Pseudosciaena crocea*). Fujian Fish. (2022) 33:58–61. doi: 10.3969/j.issn.1006-5601.2011.02.012

[6] Yang G Li X Lu W Jiang F Chen Z Zhang W. Pathological analysis of scuticociliatosis in indoor recirculating aquaculture of large yellow croaker. J Fish Res. (2023) 45:523–9. doi: 10.14012/j.cnki.fjsc.2023.06.001

[7] Chi HS Jiang Q Pan Y Ling N. Molecular identification and phylogenetic analysis of scuticociliate on large yellow croaker. Fujian J Anim Husb Vet. (2023) 45:1–6. doi: 10.3969/j.issn.1003-4331.2023.04.001

[8] Lin N Pan Y Zhan Z Xu B Gong H Zeng H. *Miamiensis avidus*, a novel scuticociliate pathogen isolated and identified from cultured large yellow croaker (*Larimichthys crocea*). Pathogens. (2024) 13:618–8. doi: 10.3390/pathogens13080618, PMID: 39204219 PMC11357409

[9] Kim SM Cho JB Kim SK Nam YK Kim KH. Occurrence of scuticociliatosis in olive flounder *Paralichthys olivaceus* by *Phiasterides dicentrarchi* (Ciliophora: Scuticociliatida). Dis Aquat Org. (2004) 62:233–8. doi: 10.3354/dao062233, PMID: 15672879

[10] Liu X Lei Y Ren Z Zhou S Qian D Yu Y. Isolation, characterization and virulence of *Mesanophrys* sp. (Ciliophora: Orchitophryidae) in farmed swimming crab (*Portunus trituberculatus*) in eastern China. J Fish Dis. (2020) 43:1419–29. doi: 10.1111/jfd.13248, PMID: 32880988

[11] Zhou RL Xie X Yin F. Isolation, characterization and virulence of *Metanophrys* sp. (Ciliophora: Scuticociliatida) from large yellow croaker (Larimichthys crocea) in China. Aquaculture. (2024) 578:740132. doi: 10.1016/j.aquaculture.2023.740132

[12] Song W Shang H Chen Z Ma H. Comparison of some closely-related Metanophrys-taxa with description of a new species Metanophrys similis nov. spec. (Ciliophora, Scuticociliatida). Euro J Protistol. (2002) 38:45–53. doi: 10.1078/0932-4739-00848

[13] Yan L-T Jiang Y Xu Q Ding G-M Chen X-Y Liu M. Reproductive dynamics of the large yellow croaker *larimichthys crocea* (Sciaenidae), A commercially important fishery species in China. Front Mar Sci. (2022) 9:868580. doi: 10.3389/fmars.2022.868580

[14] Liu X. 2024 China Fisheries Statistical Yearbook. Beijing: China Agricultural Press (2024). p. 159.

[15] Hadwan MH. Simple spectrophotometric assay for measuring catalase activity in biological tissues. BMC Biochem. (2018) 19:1–8. doi: 10.1186/s12858-018-0097-5, PMID: 30075706 PMC6091033

[16] Zeinali F Homaei A Kamrani E. Sources of marine superoxide dismutases: Characteristics and applications. Int J Biol Macromol. (2015) 79:627–37. doi: 10.1016/j.ijbiomac.2015.05.053, PMID: 26047895

[17] Mallatt J Craig CW Yoder MJ. Nearly complete rRNA genes assembled from across the metazoan animals: Effects of more taxa, a structure-based alignment, and paired-sites evolutionary models on phylogeny reconstruction. Mol Phylogen Evol. (2010) 55:1–17. doi: 10.1016/j.ympev.2009.09.028, PMID: 19786108

[18] Yenkoyan K Harutyunyan H Harutyunyan A. A certain role of SOD/CAT imbalance in pathogenesis of autism spectrum disorders. Free Rad.l Biol Med. (2018) 123:85–95. doi: 10.1016/j.freeradbiomed.2018.05.070, PMID: 29782990

[19] Lee EH Kim SM Kwon SR Kim SK Nam YK Kim KH. Comparison of toxic effects of nitric oxide and peroxynitrite on *Uronema marinum* (Ciliata: Scuticociliatida). Dis Aqua Org. (2004) 58:255–60. doi: 10.3354/dao058255, PMID: 15109150

[20] Wang X Gao Y Ni X Guo Z Zhang J Wang X. Transcriptomic analysis of gene expression in immune pathways in the spleen of Takifugu rubripes after immunization with scuticociliate vaccine. Aquaculture. (2024) 740380:581. doi: 10.1016/j.aquaculture.2023.740380, PMID: 40576689

[21] Wang PJ Yang WK Lin CH Hwang HH Lee TH. FXYD8, a novel regulator of renal na^+^/K^+^-ATPase in the euryhaline teleost, *tetraodon nigroviridi*s. Front Physiol. (2017) 8:576. doi: 10.3389/fphys.2017.00576, PMID: 28848450 PMC5550679

[22] Cheng CH Yang FF Ling RZ Liao SA Miao YT Ye CX. Effects of ammonia exposure on apoptosis, oxidative stress and immune response in pufferfish (*Takifugu obscurus*). Aqua Toxicol. (2015) 164:61–71. doi: 10.1016/j.aquatox.2015.04.004, PMID: 25917764

[23] Costa V Angelini C De Feis I Ciccodicola A. Uncovering the complexity of transcriptomes with RNA-Seq. J Biomed Biotech. (2010) 2010:853916. doi: 10.1155/2010/853916, PMID: 20625424 PMC2896904

[24] You C Okano H Hui S Zhang Z Kim M Gunderson CW. Coordination of bacterial proteome with metabolism by cyclic AMP signalling. Nature. (2013) 500:301–6. doi: 10.1038/nature12446, PMID: 23925119 PMC4038431

[25] Valle A Leiro JM Pereiro P Figueras A Novoa B Dirks RPH. Interactions between the Parasite *Philasterides dicentrarchi* and the Immune System of the Turbot *Scophthalmus maximus.* A Transcriptomic Analysis. Biology-Basel. (2020) 9:337. doi: 10.3390/biology9100337, PMID: 33076342 PMC7602577

[26] Makowski L Chaib M Rathmell JC. Immunometabolism: From basic mechanisms to translation. Immunol Rev. (2020) 295:5–14. doi: 10.1111/imr.12858, PMID: 32320073 PMC8056251

[27] Chen Y Chen Y Shi C Huang Z Zhang Y Li S. SOAPnuke: a MapReduce acceleration-supported software for integrated quality control and preprocessing of high-throughput sequencing data. Gigascience. (2018) 7:1–6. doi: 10.1093/gigascience/gix120, PMID: 29220494 PMC5788068

[28] Kim D Langmead B Salzberg SL. HISAT: A fast spliced aligner with low memory requirements. Nat Methods. (2015) 12:357-360. doi: 10.1038/nmeth.3317, PMID: 25751142 PMC4655817

[29] Langmead B. Aligning Short Sequencing Reads with Bowtie. Current protocols in bioinformatics. Curr Protoc Bioinform. (2010) Chapter 11:Unit 11.17. doi: 10.1002/0471250953.bi1107s32, PMID: 21154709 PMC3010897

[30] Dewey CN Bo L. RSEM: accurate transcript quantification from RNA-Seq data with or without a reference genome. BMC Bioinf. (2011) 12:323–3. doi: 10.1186/1471-2105-12-323, PMID: 21816040 PMC3163565

[31] Love MI Huber W Anders S. Moderated estimation of fold change and dispersion for RNA-seq data with DESeq2. Genome Biol. (2014) 15:550. doi: 10.1186/s13059-014-0550-8, PMID: 25516281 PMC4302049

[32] Charlie-Silva I Feitosa NM Pontes LG Fernandes BH Nóbrega RH Gomes JMM. Plasma proteome responses in zebrafish following λ-carrageenan-Induced inflammation are mediated by PMN leukocytes and correlate highly with their human counterparts. Front Immunol. (2022) 13:1019201. doi: 10.3389/fimmu.2022.1019201, PMID: 36248846 PMC9559376

[33] Guirao B Joanny JF. Spontaneous creation of macroscopic flow and metachronal waves in an array of cilia. Biophys J. (2007) 92:1900–17. doi: 10.1529/biophysj.106.084897, PMID: 17189311 PMC1861806

[34] Leiro J Arranz JA Iglesias R Ubeira FM Sanmartín ML. Effects of the histiophagous ciliate *Philasterides dicentrarchi* on turbot phagocyte responses. Fish Shellfish Immunol. (2004) 17:27–39. doi: 10.1016/j.fsi.2003.11.003, PMID: 15145415

[35] Piazzon MC Wiegertjes GF Leiro J Lamas J. Turbot resistance to *Philasterides dicentrarchi* is more dependent on humoral than on cellular immune responses. Fish Shellfish Immunol. (2011) 30:1339–47. doi: 10.1016/j.fsi.2011.02.026, PMID: 21420498

[36] Zhang K Zhang W Li R Lu J Chen Q Hu H. Dynamic Distribution of *Mesanophrys* sp. and Tissue Enzyme Activities in Experimentally Infected Mud Crab *Scylla paramamosain* . Fishes. (2023) 8:249. doi: 10.3390/fishes8050249

[37] Rizzo M. Measurement of malondialdehyde as a biomarker of lipid oxidation in fish. Am J Analytical Chem. (2024) 15:303–32. doi: 10.4236/ajac.2024.159020

[38] Mahdy OA Abdel-Maogood SZ Abdelsalam M Salem MA. A multidisciplinary study on Clinostomum infections in Nile tilapia: micro-morphology, oxidative stress, immunology, and histopathology. BMC veterinary Res. (2024) 20:60. doi: 10.1186/s12917-024-03901-7, PMID: 38378547 PMC10877748

[39] Bai H Zhou T Zhao J. Transcriptome analysis reveals the temporal gene expression patterns in skin of large yellow croaker (*Larimichthys crocea*) in response to *Cryptocaryon irritans* infection. Fish Shellfish Immunol. (2020) 99):462–72. doi: 10.1016/j.fsi.2020.02.024, PMID: 32070786

[40] Martínez I Oliveros JC Cuesta I de la Barrera J Ausina V Casals C. Apoptosis, toll-like, RIG-I-like and NOD-like receptors are pathways jointly induced by diverse respiratory bacterial and viral pathogens. Front Microbiol. (2017) 8:276. doi: 10.3389/fmicb.2017.00276, PMID: 28298903 PMC5331050

[41] Palti Y. Toll-like receptors in bony fish: From genomics to function. Dev Comp Immunol. (2011) 35:1263–72. doi: 10.1016/j.dci.2011.03.006, PMID: 21414346

[42] Vasta GR Nita-Lazar M Giomarelli B Ahmed H Du S Cammarata M. Structural and functional diversity of the lectin repertoire in teleost fish: Relevance to innate and adaptive immunity. Dev Comp Immunol. (2011) 35:1388–99. doi: 10.1016/j.dci.2011.08.011, PMID: 21896283 PMC3429948

[43] Xie J Hodgkinson JW Katzenback BA Kovacevic N Belosevic M. Characterization of three Nod-like receptors and their role in antimicrobial responses of goldfish (*Carassius auratus* L.) macrophages to *Aeromonas salmonicida* and *Mycobacterium marinum* . Dev Comp Immunol. (2013) 39:180–7. doi: 10.1016/j.dci.2012.11.005, PMID: 23194927

[44] Paredes LC Camara NOS Braga TT. Understanding the metabolic profile of macrophages during the regenerative process in zebrafish. Front Physiol. (2019) 10:617. doi: 10.3389/fphys.2019.00617, PMID: 31178754 PMC6543010

[45] Song J Li Z Zhou L Chen X Qi W Sew G. FOXO-regulated OSER1 reduces oxidative stress and extends lifespan in multiple species. Nat Commun. (2024) 15:7144. doi: 10.1038/s41467-024-51542-z, PMID: 39164296 PMC11336091

[46] Kousteni S. FoxO1, the transcriptional chief of staff of energy metabolism. Bone. (2012) 50:437–43. doi: 10.1016/j.bone.2011.06.034, PMID: 21816244 PMC3228887

[47] Gupta P Srivastav S Saha S Das PK Ukil A. *Leishmania donovani* inhibits macrophage apoptosis and pro-inflammatory responses through AKT-mediated regulation of β-catenin and FOXO-1. Cell Death Dif. (2016) 23:1815–26. doi: 10.1038/cdd.2016.101, PMID: 27662364 PMC5071566

[48] Adderley JD Freyend J Jackson SA Bird M Burns AL Anar B. Analysis of erythrocyte signalling pathways during *Plasmodium falciparum* infection identifies targets for host-directed animalaria intervention. Nat Commun. (2020) 11:4015. doi: 10.1038/s41467-020-17829-7, PMID: 32782246 PMC7419518

[49] Ullah I Barrie U Kernen RM Mamula ET Khuong FTH Booshehri LM. Src- and Abl-family kinases activate spleen tyrosine kinase to maximize phagocytosis and Leishmania infection. J Cell Sci. (2023) 136:jcs260809. doi: 10.1242/jcs.260809, PMID: 37357611 PMC10399977

[50] Bock FJ Riley JS. When cell death goes wrong: inflammatory outcomes of failed apoptosis and mitotic cell death. Cell Death Dif. (2023) 30:293–303. doi: 10.1038/s41418-022-01082-0, PMID: 36376381 PMC9661468

[51] Lamour SD Veselkov KA Posma JM Rogers ME Croft S Marchesi JR. Metabolic, immune, and gut microbial signals mount a systems response to *Leishmania major* infection. J Prot Res. (2015) 14:318–29. doi: 10.1021/pr5008202, PMID: 25369177

[52] Galindo-Villegas J García-Moreno D de Oliveira S Meseguer J Mulero V. Regulation of immunity and disease resitance by commensal microbes and chromatin modifications during zebrafish development. PNAS. (2012) 109:E605–14. doi: 10.1073/pnas.1209920109, PMID: 22949679 PMC3465450

[53] Kelly MN Kolls JK Happel K Schwartzman JD Schwarzenberger P Combe C. Interleukin-17/Interleukin-17 Receptor-Mediated Signaling Is Important for Generation of an Optimal Polymorphonuclear Response against *Toxoplasma gondii* Infection. Infection Immunity. (2005) 73:617–21. doi: 10.1128/IAI.73.1.617-621.2005, PMID: 15618203 PMC538931

[54] Ribeiro CMS Pontes MJSL Bird S Chadzinska M Scheer M Verburg-van Kemenade BML. Trypanosomiasis-induced th17-like immune responses in carp. PloS One. (2010) 5:e13012. doi: 10.1371/journal.pone.0013012, PMID: 20885956 PMC2946394

[55] Ding Y Ai C Mu Y Ao J Chen X. Molecular characterization and evolution analysis of five interleukin-17 receptor genes in large yellow croaker (*Larimichthys crocea*). Fish Shellfish Immunol. (2016) 58:332–9. doi: 10.1016/j.fsi.2016.09.017, PMID: 27633682

[56] Peck A Mellins ED. Precarious balance: th17 cells in host defense. Infection Immunity. (2010) 78:32. doi: 10.1128/IAI.00929-09, PMID: 19901061 PMC2798221

[57] Wang T Zhou N Ding F Hao Z Galindo-Villegas J Du Z. Xylanase enhances gut microbiota-derived butyrate to exert immune-protective effects in a histone deacetylase-dependent manner. Microbiome. (2024) 12:212. doi: 10.1186/s40168-024-01934-6, PMID: 39434145 PMC11492574

[58] He Y Zhu W Xu T Liao Z Su J. Identification and immune responses of thrombocytes in bacterial and viral infections in grass carp (*Ctenopharyngodon idella*). Fish Shellfish Immunol. (2022) 123:314–23. doi: 10.1016/j.fsi.2022.03.009, PMID: 35306178

[59] Stosik M Tokarz-Deptuła B Deptuła W. Characterisation of thrombocytes in osteichthyes. J Vet Res. (2019) 63:123–31. doi: 10.2478/jvetres-2019-0017, PMID: 30989144 PMC6458560

[60] Kang H Liang QJ Hu R Li ZH Liu Y Wang WN. Integrative mRNA-miRNA interaction analysis associated with the immune response of *Epinephelus coioddes* to *Vibrio alginolyticus* infection. Fish Shellfish Immunol. (2019) 90:404–12. doi: 10.1016/j.fsi.2019.05.006, PMID: 31077847

[61] Yazhou H Anxing L Yang X Biao J Geling L Xiaochun L. Transcriptomic variation of locally-infected skin of *Epinephelus coioides* reveals the mucosal immune mechanism against *Cryptocaryon irritans* . Fish Shellfish Immunol. (2017) 66:398–410. doi: 10.1016/j.fsi.2017.05.042, PMID: 28526573

[62] Dezfuli BS Lorenzoni M Carosi A Giari L Bosi G. Teleost innate immunity, an intricate game between immune cells and parasites of fish organs: who wins, who loses. Front Immunol. (2023) 14:1250835. doi: 10.3389/fimmu.2023.1250835, PMID: 37908358 PMC10613888

[63] Molnar N Miskolci V. Imaging immunometabolism in situ in live animals. Immunometabolism. (2024) 6:e00044 742721. doi: 10.1097/IN9.0000000000000044, PMID: 39296471 PMC11406703

[64] Pålsson-McDermott E O’Neill LAJ. Targeting immunometabolism as an anti-inflammatory strategy. Cell Res. (2020) 30:300–14. doi: 10.1038/s41422-020-0291-z, PMID: 32132672 PMC7118080

[65] Galindo-Villegas J Montalban-Arques A Liarte S de Oliveira S Pardo-Pastor C Rubio-Moscardo F. TRPV4-mediated detection of hyposmotic stress by skin keratinocytes activates developmental immunity. J Immunol. (2016) 196:738–49. doi: 10.4049/jimmunol.1501729, PMID: 26673139

[66] Munang’andu HM Galindo-Villegas J David L. Teleosts genomics: progress and prospects in disease prevention and control. Int J Mol Sci. (2018) 19:1083. doi: 10.3390/ijms19041083, PMID: 29617353 PMC5979277

[67] Paramá A Iglesias R Alvarez MF Leiro J Ubeira MF UF. and Sanmartín ML. Cysteine proteinase activities in the fish pathogen *Philasterides dicentrarchi* (Ciliophora: Scuticociliatida). Parasitology. (2004) 128:541–8. doi: 10.1017/S0011382004004883, PMID: 15180322

[68] Lee EH Kim CS Cho JB Ahn KJ Kim KH. Measurement of protease activity of live Uronema marinum (Ciliata: Scuticociliatida) by fluorescence polarization. Dis Aquat Organ. (2003) 54:85–8. doi: 10.3354/dao054085, PMID: 12718476

