# Supplementary material for: Integrated transcriptomic and immune enzymatic analyses uncover coordinated immunometabolic responses in large yellow croaker (Larimichthys crocea) to Metanophrys sp. infection
Source: Front Immunol. 2025 Jul 16;16:1636453. doi: 10.3389/fimmu.2025.1636453 (PMC12307142; doi:10.3389/fimmu.2025.1636453)
Supplement: Supplementary file 1 [file Table1.docx]

**Supplementary Table 1.** Primer sets used for qPCR

| **Gene  (name)** | **Gene  (abbreviation)** | **Primers （5′-3′）** | **Amplicon size  (bp)** |
| --- | --- | --- | --- |
| Protein phosphatase 3, regulatory  subunit B | *ppp3r* | F - GGACGAGTGTAGTTAGCGG | 240 |
|  |  | R - TGCCCAATCTTTATTTCTG |  |
| Mucosa-associated lymphoid tissue  lymphoma translocation protein 1 | *malt1* | F - AGTAGACGCACCAAACCCG | 280 |
|  |  | R - CCGTAACCCTCAGGACAAT |  |
| p38 mitogen-activated protein kinase | *p38* | F - ACTTCTTCCCGTAGTCCAG | 209 |
|  |  | R - TTCTAACCCAACTTTCCCT |  |
| Caspase-8 | *casp8* | F - AAGATGCTCCCTAATGAAA | 172 |
|  |  | R - TCTACACGCTGACCTGAAT |  |
| Mitogen-activated protein kinase  kinase kinase 14 | *map3k14* | F - GAATCTCCTCCACCAACGG | 261 |
|  |  | R - GGTCACCAGTCCAAATCCC |  |
| Signal transducer and activator of  transcription 1 | *stat1* | F - ACAGGCAAATACAGTAACCA | 287 |
|  |  | R - CAAAGAGGCTCCACTAACA |  |
| Lipopolysaccharide-binding protein | *lbp* | F - GAGATTCAACAGTTCCCACAA | 291 |
|  |  | R - ACAGAGGACCATCGGCTAA |  |
| Nuclear factor of activated T-cells,  cytoplasmic 3 | *nfatc3* | F - TTTTACCGCACAAGATTAG | 195 |
|  |  | R - AAACATACTTTAGCCACCT |  |
| CD8 alpha chain | *cd8a* | F - CATCTTACAGCGTCGTTTC | 222 |
|  |  | R - ACATTCATTTGGGATTTGG |  |
| Fas-associated protein with death  domain | *fadd* | F - CACCTGGATGGAGACTGAC | 160 |
|  |  | R - GGACAAGCCTAATGGAACT |  |
| Nuclear factor of activated T-cells,  cytoplasmic 2 | *nfatc2* | F - CCATCTCGCACCATTCTTA | 131 |
|  |  | R - TTACCAGCAGGAGTCACCA |  |
| Interleukin-8 | *il8* | F - GTCAAAGACAGCGACAATA | 179 |
|  |  | R - ACTTCACTAACTGCCTCCT |  |
| Beta-actin | *β-actin* | F - GTTGTAGTATCGCTTGTATGT | 239 |
|  |  | R - TTGGGATTGTTTAGTCAGT |  |
